# Repeated glucose spikes and insulin resistance synergistically deteriorate endothelial function and bardoxolone methyl ameliorates endothelial dysfunction

**DOI:** 10.1371/journal.pone.0263080

**Published:** 2022-01-24

**Authors:** Kazuma Ogiso, Sigfrid Casmir Shayo, Shigeru Kawade, Hiroshi Hashiguchi, Takahisa Deguchi, Yoshihiko Nishio

**Affiliations:** Department of Diabetes and Endocrine Medicine, Kagoshima University Graduate School of Medical and Dental Science, Kagoshima, Japan; Max Delbruck Centrum fur Molekulare Medizin Berlin Buch, GERMANY

## Abstract

**Background:**

Both insulin resistance and postprandial glucose spikes are known for their potential to induce vascular endothelial dysfunction in individuals with metabolic syndrome. However, these factors are inextricable, and therefore, their relative contributions to inducing endothelial dysfunction remain elusive. In this study, we aimed to disentangle the effects of these factors and clarify whether bardoxolone methyl (CDDO-Me), a novel nuclear factor erythroid 2-related factor 2 (Nrf2) activator, protects against glucose spike-induced endothelial dysfunction.

**Methods:**

We induced glucose spikes twice daily for a duration of 1 week to rats fed a standard/control diet (CD) and Western-type diet (WTD). Endothelium-dependent relaxation (EDR) was evaluated using isolated thoracic aortas. Gene expression and dihydroethidium (DHE)-fluorescence studies were carried out; the effect of CDDO-Me on aortic endothelial dysfunction in vivo was also evaluated.

**Results:**

Neither WTD-induced insulin resistance nor pure glucose spikes significantly deteriorated EDR. However, under high-glucose (20 mM) conditions, the EDR of thoracic aortas of WTD-fed rats subjected to glucose spikes was significantly impaired. In this group of rats, we observed significantly enhanced DHE fluorescence as a marker of reactive oxygen species, upregulation of an oxidative stress-related gene (NOX2), and downregulation of an antioxidant gene (SOD2) in the thoracic aortas. As expected, treatment of the thoracic aorta of this group of rats with antioxidant agents significantly improved EDR. We also noted that pretreatment of aortas from the same group with CDDO-Me attenuated endothelial dysfunction, accompanied by a correction of the redox imbalance, as observed in gene expression and DHE fluorescence studies.

**Conclusions:**

For the first time, we showed that insulin resistance and glucose spikes exert a synergistic effect on aortic endothelial dysfunction. Furthermore, our study reveals that CDDO-Me ameliorates endothelial dysfunction caused by glucose spikes in a rat model of metabolic syndrome.

## Introduction

Cardiovascular disease (CVD) is currently the leading cause of adult morbidity and mortality worldwide [1]. In this modern era, metabolic syndrome, which is characterized by abdominal obesity and insulin resistance, among others, is the main culprit responsible for atherogenesis and subsequent development of CVD [2]. Postprandial hyperglycemia, also known as a glucose spike (GS), is associated with insulin resistance. Similar to insulin resistance, GS is now known as a robust determinant of the CVD risk [3]. According to previous studies, glucose levels measured after an oral glucose tolerance test (OGTT) are more strongly associated with the carotid intima-media thickness (cIMT), a surrogate marker of atherosclerosis, than the levels of fasting plasma glucose (FPG) or hemoglobin A1c (HbA1c) [4]. Even more remarkably, GS is a strong predictor of CVD even in nondiabetic individuals and in individuals within the nondiabetic glucose range [5].

Endothelial dysfunction, namely, damage to the vascular endothelium characterized by impaired flow-mediated endothelium-dependent vasodilation (FMD), is central to the process of atherogenesis. GS is known to rapidly suppress flow-mediated endothelium-dependent vasodilation (FMD) by inducing the production of reactive oxygen species (ROS) [6,7]. Moreover, GS increases the adhesion of monocytes responsible for vascular inflammation to the arterial endothelium [8]. The ability of GS to enhance atherogenic lesion formation in rats fed an atherogenic diet further corroborates the role of GS in atherogenesis [9]. Surprisingly, despite the available experimental evidence for the role of GS in the development of atherogenic lesions, evidence from clinical trials conducted thus far has not yielded consistent results. Both prandial (rapid-acting) insulin and nateglinide, which are agents that target postprandial hyperglycemia, do not reduce CVD events (the HEART2D and NAVIGATOR trials) [10,11]. Even acarbose, an α-glucosidase inhibitor, does not reduce the risk of major adverse cardiovascular events (ACE trial) [12]. Although the explanations for these findings have yet to be explored, other risk factors related to insulin resistance, such as dyslipidemia (including postprandial hypertriglyceridemia [13,14]) or hyperinsulinemia, may additively contribute to endothelial dysfunction. Therefore, further experimental studies are warranted to discern the independent role of GS in endothelial dysfunction.

A review of previous reports related to GS shows that oxidative stress is the chief driver of endothelial dysfunction. Therefore, strategies targeting oxidative stress may be a useful approach for ameliorating endothelial dysfunction induced by GS and possibly other metabolic factors related to insulin resistance, such as hyper-free fatty acidemia. Recently, the nuclear factor erythroid 2-related factor 2 (Nrf2) activator bardoxolone methyl (CDDO-Me) has been shown to exert a renal-protective effect on individuals with diabetic nephropathy, mainly through antioxidant and anti-inflammatory effects (TSUBAKI study) [15]. Moreover, Nrf2 activators also preserve endothelial function in animal models of chronic kidney disease (CKD) and diabetes [16,17]. Based on these results, CDDO-Me may have the potential to treat GS-induced endothelial dysfunction by attenuating oxidative stress.

In this study, we aimed to investigate the individual and combined effects of GS and insulin resistance on endothelial dysfunction. We also explored the conditions in which endothelial function is most vulnerable to GS and evaluated whether CDDO-Me prevents GS-induced endothelial dysfunction.

## Methods

### Animal studies

Seven-week-old male Wistar rats were obtained from KBT Oriental Co., Ltd. (Saga, Japan) and housed at 23 ± 1°C on a 12-h light/12-h dark cycle with ad libitum access to food and water. After a 1-week acclimation period, the rats were fed a control diet (CD) or a Western-type diet (WTD) for 13 weeks (8–21 weeks old) according to their group. The CD contained 4.7% of calories from fat, 23.3% of calories from protein, and 55.6% of calories from carbohydrates (3.6 kcal/g; MF, Oriental Yeast Co., Ltd., Tokyo, Japan), and the WTD contained 39.9% of calories from fat, 15% of calories from protein, and 44.3% of calories from carbohydrates (4.5 kcal/g; F2WTD, Oriental Yeast Co., Ltd.). The WTD group of rats was allowed access to feed ad libitum, and the CD group of rats was pair-fed (limited to the amount of food consumed by the rats in the WTD group) to prevent obesity due to excess food intake.

In the first cohort, the rats were assigned to one of four groups (N = 7 rats per group) according to the factor of diet or glucose spike (GS): 1) CD-GS (-), control diet but no glucose spike; 2) CD-GS (+), control diet and glucose spike; 3) WTD-GS (-), Western-type diet but no glucose spike; and 4) WTD-GS (+), Western-type diet and glucose spike. The CD group represented nonobese model rats without insulin resistance, and the WTD group represented obese model rats with insulin resistance. At 20 weeks of age, rats in the GS (-) and GS (+) groups were intraperitoneally injected with saline (5 mL/kg) and 20% glucose (1 g/5 mL/kg body weight [BW]), respectively, for one week twice daily at approximately 08:00 a.m. and 04:00 p.m. (see S1A Fig for details). In the second cohort, four groups from the first cohort were additionally divided into vehicle and CDDO-Me groups (N = 4 rats per group). At 19 weeks of age, rats in the vehicle and CDDO-Me groups were orally administered vehicle (sesame oil, S3547, Sigma-Aldrich, St. Louis, MO, USA) or CDDO-Me (3 mg/kg BW; SMB00376, Sigma-Aldrich, St. Louis, MO, USA), which was solubilized in sesame oil, for two weeks once daily (see S1B Fig for details). At 20 weeks of age, rats in the GS (-) and GS (+) groups were intraperitoneally injected with saline and 20% glucose, respectively, twice daily for one week as described for the first cohort.

Blood samples and thoracic aortas were collected from the first and second cohorts at 21 weeks of age after a 14-hour overnight fast. All rats were anesthetized with isoflurane (5% induction and 3% maintenance inhaled), and thoracotomy was performed under anesthesia. After collecting blood samples from the heart, rats were euthanized by exsanguination and perfused with heparinized saline. Thoracic aortas were then isolated rapidly, and epididymal, retroperitoneal, mesenteric fat pads and livers were harvested and weighed. Serum or plasma parameters were measured with a Rat Insulin ELISA (Morinaga Institute of Biological Science, Inc., Yokohama, Japan), LabAssay Triglyceride assay (TG; FUJIFILM Wako Pure Chemical Corporation, Osaka, Japan), LabAssay NEFA assay (FFAs, free fatty acids; FUJIFILM Wako), LabAssay Cholesterol assay (TC, total cholesterol; FUJIFILM Wako), Rat Tumor Necrosis Factor α (TNFα) Quantikinase ELISA (R&D Systems, Inc., McKinley Place, MN, USA) and Glucose Assay Kit II (Funakoshi Co., Ltd., Tokyo, Japan). Visceral fat mass was evaluated as the adiposity index, which was defined as the ratio of epididymal, retroperitoneal and mesenteric fat grams to body weight [18]. Insulin resistance was assessed by performing an insulin tolerance test (ITT) and homeostasis model assessment of insulin resistance (HOMA-IR). For the ITT, rats were injected intraperitoneally with 0.5 IU/kg BW regular human insulin (Humulin R, Eli Lilly Japan K.K., Kobe, Japan), and insulin resistance was evaluated as the decreasing blood glucose area under the curve (AUC). HOMA-IR was calculated with the formula HOMA IR = serum insulin (mmol/L)*(blood glucose (mmol/L)/22.*5* [19].

All animal protocols were reviewed and approved by the Laboratory Animal Committees of Kagoshima University Graduate School and were performed in accordance with the guidelines for the care and use of laboratory animals (approval number: MD20086). All efforts were made to minimize animal suffering and to use the minimal number of animals necessary to produce reliable results.

### Continuous interstitial glucose monitoring

When starting saline or glucose administration at 20 weeks of age, a FreeStyle Libre Pro^®^ sensor (Abbott Diabetes Care, IL, USA), which continuously records interstitial glucose levels every fifteen minutes, was attached to the backs of the rats to record interstitial glucose levels for two days. After removing the sensor, the data were extracted using FreeStyle Libre Pro software (Abbott Diabetes Care). A GS was defined as an increased interstitial glucose level above 5 mM, which was the difference between baseline and peak interstitial glucose levels. Although interstitial glucose levels reflect blood glucose levels, they physiologically lag 5 minutes behind blood glucose levels and tend to be lower than blood glucose levels, especially when blood glucose levels are high [20].

### Vascular reactivity

After animals were euthanized at 21 weeks of age, each thoracic aorta was rapidly isolated, carefully cleared of perivascular fat and adventitia and placed in oxygenated physiological saline solution (PSS; 130 mmol/L NaCl, 14.9 mmol/L NaHCO_3_, 4.7 mmol/L KCl, 1.18 mmol/L KH_2_PO_4_, 1.17 mmol/L MgSO_4_^-^7H_2_O, 1.6 mmol/L CaCl_2_^-^2H_2_O, 0.026 mmol/L EDTA, and 5.5 mmol/L glucose [pH 7.4]). Two rings (2 mm long) were cut from each thoracic aorta per rat, mounted onto a Multiwire Myograph System 620M (Danish Myo Technology, Aarhus, Denmark) and perfused through the chambers with 5% CO_2_ and 95% O_2_ at 37°C. Changes in isometric tension were measured using a LabChart Pro data acquisition system (ADInstruments Pty Ltd., Castle Hill, Australia) as previously described [21]. After 30 minutes of equilibration, the aortic rings were exposed to a high-potassium physiological saline solution (KPSS) (74.7 mmol/L NaCl, 14.9 mmol/L NaHCO_3_, 60 mmol/L KCl, 1.18 mmol/L KH_2_PO_4_, 1.17 mmol/L MgSO_4_^-^7H_2_O, 1.6 mmol/L CaCl_2_^-^2H_2_O, 0.026 mmol/L EDTA, and 5.5 mmol/L glucose) to assess the maximal tension. After washout, one of the two samples collected from each rat was equilibrated in a normal-glucose PSS chamber (5.5 mmol/L glucose PSS [Glu 5.5 mM]), and the other was equilibrated in a high-glucose PSS chamber (20 mmol/L glucose PSS [Glu 20 mM]) for 2 hours. After an equilibration period, endothelium-dependent relaxation (EDR) was determined by cumulatively adding acetylcholine (ACh; 3.2 nmol/L-100 μmol/L) (NACALAI TESQUE, INC., Kyoto, Japan) to phenylephrine (PE; ~1 μmol/L) (Sigma-Aldrich Japan Co., LLC, Tokyo, Japan)-precontracted segments. Aortas equilibrated in a normal-glucose PSS chamber were also equilibrated in raffinose (20 mmol/L) (FUJIFILM Wako Pure Chemical Corporation, Osaka, Japan) for 2 hours as an osmotic control for 20 mM glucose. Sodium nitroprusside (SNP; 100 pmol/L-100 μmol/L) (Sigma-Aldrich Japan Co., LLC), an exogenous nitric oxide (NO) donor, was used to test endothelium-independent relaxation. Maximal relaxation was induced by papaverine (PPV; 100 μmol/L) (Nichi-Iko Pharmaceutical Co., Ltd., Toyama, Japan). Endothelial integrity was tested by measuring ACh-induced relaxation (100 μmol/L) in segments previously contracted with a concentration of PE (~1 μmol/L) that induced 50–70% of the contraction induced by 60 mmol/L KPSS. Relaxation in response to acetylcholine greater than 50% was considered to indicate endothelial functional integrity.

In some experiments, the role of free radicals in EDR was evaluated by incubating aortic rings with the following agents for 30 minutes before the EDR measurement: indomethacin (10 μmol/L), a prostaglandin synthetase inhibitor (FUJIFILM Wako Pure Chemical Corporation) [22]; allopurinol (100 μmol/L), a xanthine oxidase inhibitor (Sigma-Aldrich Japan Co., LLC) [23]; insulin (10 nmol/L), Humulin R (Eli Lilly Japan K.K., Kobe, Japan) [24]; apocynin (100 μmol/L), an NADPH oxidase (NOX) inhibitor (Tokyo Chemical Industry Co., Ltd., Tokyo, Japan) [23]; GKT137831 (10 μmol/L), a NOX1 and 4 inhibitor (Cayman Chemical, MI, USA) [25]; GSK2795039 (25 μmol/L), a NOX2 inhibitor (MedChemExpress, NJ, USA) [26]; superoxide dismutase (SOD) (150 U/mL), a superoxide scavenger (Sigma-Aldrich Japan Co., LLC) [27]; catalase (6,250 U/mL), a hydrogen peroxide scavenger (Sigma-Aldrich Japan Co., LLC) [28]; Mito-TEMPO (100 nmol/L), a mitochondria-targeted superoxide scavenger (Funakoshi Co., Ltd., Tokyo, Japan) [29]; and Mn(III) tetra(4-benzoic acid) porphyrin chloride (MnTABP) (10 μmol/L), a SOD mimetic and peroxynitrite scavenger (Funakoshi Co., Ltd.) [30]. L-N^G^-nitro-arginine methyl ester (L-NAME) (100 μmol/L) and a nitric oxide synthase (NOS) inhibitor (FUJIFILM Wako Pure Chemical Corporation) [31] were also used as pretreatments to eliminate EDR.

Relaxation induced by ACh and SNP is reported as the percentage of the maximum relaxation obtained with PPV. For each concentration-response curve, pD2 (log of the half-maximal effective concentration [EC50]) was calculated using a nonlinear regression analysis with the statistical software R version 3.6.1 (The R Foundation for Statistical Computing, Vienna, Austria).

### Quantitative real-time PCR

The excised thoracic aortas were frozen in liquid nitrogen, pulverized in a mill, and then homogenized with a POLYTRON PT 2500 E (Kinematica AG, Luzern, Schweiz). Homogenized tissues were lysed with 900 μL of QIAzol Lysis Reagent (QIAGEN K.K., Tokyo, Japan). Genomic DNA contamination of the aqueous phase was reduced with gDNA Eliminator Solution (QIAGEN K.K.). After separating the phases with the addition of 180 μL of chloroform, RNA was isolated using an RNeasy Mini Kit (QIAGEN K.K.). An Applied Biosystems High-Capacity cDNA Reverse Transcription Kit with RNase Inhibitor (Thermo Fisher Scientific K.K., Tokyo, Japan) was used to synthesize cDNAs from 1 μg of total RNA. Quantitative PCR was performed using an Applied Biosystems StepOnePlus Real-Time PCR System with TaqMan™ Fast Universal PCR Master Mix (Thermo Fisher Scientific K.K.). Relative gene expression was calculated using the ΔΔCt method. Gene expression was normalized to GAPDH. The primers and probes used in the present study are listed in S1 Table (Thermo Fisher Scientific K.K.).

### Oxidative stress assessment

We measured the dihydroethidium (DHE) fluorescence intensity in aortas and urinary 8-hydroxy-2-deoxyguanosine (8-OHdG) levels to evaluate local and systemic oxidative stress. Reactive oxygen species (ROS) production in the en face endothelium of rat aortas was measured with DHE (Sigma-Aldrich Japan Co., LLC) under a fluorescence microscope [32]. Briefly, the aortic rings were embedded vertically into OCT compound (Funakoshi Co., Ltd.) and immediately placed on crushed dry ice for freezing. Once frozen, 8 μm-thick sections were cut using a cryostat at -20°C. After rinsing the slides with pure H_2_O for 30 seconds to wash out the OCT compound, slides were immediately placed in a 5 μM DHE staining solution and incubated for 20 minutes at room temperature in the dark. Some sections were preincubated for 30 minutes with 250 U/mL polyethylene glycol-superoxide dismutase (PEG-SOD) (Sigma-Aldrich Japan Co., LLC) before the incubation with DHE. The DHE fluorescence intensity was measured with a fluorescence microscope (EVOS FL Auto 2 Imaging System; Thermo Fisher Scientific K.K.) at an excitation wavelength of 542 nm and an emission wavelength of 593 nm (EVOS Light Cube, RFP; Thermo Fisher Scientific K.K.). The background autofluorescence of elastin was measured at an excitation wavelength of 482 nm and an emission wavelength of 524 nm (EVOS Light Cube, RFP; Thermo Fisher Scientific K.K.) and subtracted from the fluorescence intensity of DHE. The DHE fluorescence intensity was evaluated with ImageJ software (version 1.51) (Rasband, W.S., U.S. National Institutes of Health, Bethesda, MD, USA) [33] and is presented as fold changes in fluorescence intensity relative to that of the control.

In some experiments, the ROS source was evaluated by incubating aortic rings, which were equilibrated in a high-glucose (20 mM) PSS chamber for 2 hours, with the following agents for 30 minutes before embedding, similar to the vascular reactivity experiment: apocynin (100 μmol/L), GKT137831 (10 μmol/L), GSK2795039 (25 μmol/L), Mito-TEMPO (100 nmol/L), SOD (150 U/mL), MnTABP (10 μmol/L) and catalase (6,250 U/mL).

In the second cohort, urinary 8-OHdG levels were also assessed using a commercially available ELISA kit (Cayman Chemical, Ann Arbor, MI, USA) to evaluate systemic oxygen free radical levels. Measurements are reported relative to urinary creatinine levels.

### Statistical analysis

Values are presented as the mean ± SEM. Statistical significance was determined using one-way ANOVA to compare differences between groups. When diet and GS interaction effects were evaluated as dependent variables, two-way between-group ANOVA was used; a significant interaction was interpreted by performing a subsequent simple-effects analysis with the Bonferroni correction. Dunnett’s test was used for comparisons with the control group, and differences between vehicle and CDDO-Me were evaluated using the Bonferroni correction. Concentration-response curves and body weight curves were analyzed using one-way or two-way repeated-measures ANOVA followed by the Bonferroni post hoc test. Univariate regression analysis using Pearson’s correlation coefficients was performed to assess significant associations between metabolic parameters and pD2. The differences between groups were considered significant when *P* < 0.05. All data were analyzed with R version 3.6.1 statistical software (The R Foundation for Statistical Computing, Vienna, Austria).

## Results

### The GS model was confirmed with continuous interstitial glucose monitoring

**Fig 1:**
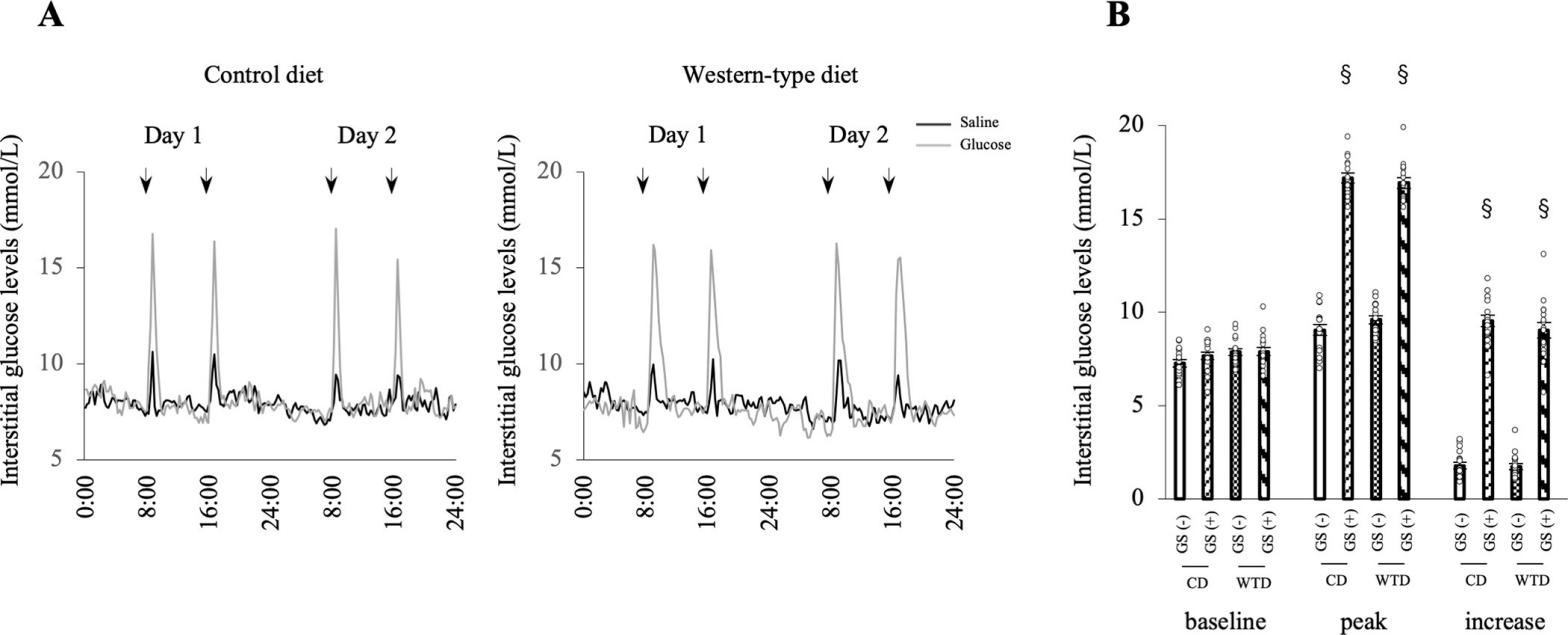
GSs are induced by intraperitoneal glucose administration. A: Representative profiles of interstitial glucose levels in four groups: GS (-), black; GS (+), gray. Interstitial glucose levels were continuously recorded with a Libre® monitor for 48 hours. Arrows indicate the time of saline or glucose administration. B: Baseline, peak and increase in interstitial glucose levels. § *P* < 0.001 compared with the GS group, two-way ANOVA. No interaction was detected between diet and GS factors. The data are presented as the means ± SEM. N = 7 rats per group. CD, control diet; WTD, Western-type diet; GS, glucose spike. The GS (-) group consisted of rats administered saline, and the GS (+) group consisted of rats administered glucose.

Interstitial glucose levels were continuously recorded with a Libre^®^ monitor for 48 hours from the 2nd day of administration of saline or glucose to confirm the glucose profile in the model rats. All incremental increases in interstitial glucose levels were > 5 mM in both the CD-GS (+) and WTD-GS (+) groups after glucose administration and were < 5 mM in both the CD-GS (-) and WTD-GS (-) groups after saline administration (Fig 1A and 1B). The small GSs observed in the GS (-) groups were presumed to be a stress response when saline was intraperitoneally administered. A significant main effect of GS was observed on both peak and incremental interstitial glucose levels (F [1, 12] = 1619.1, *P* < 0.001; F [1, 12] = 513.7, *P* < 0.001, respectively) without a diet/GS interaction, although the main effect of diet on those parameters was not significant.

### Short-term repeated GSs deteriorated EDR in diet-induced obese rats

**Fig 2:**
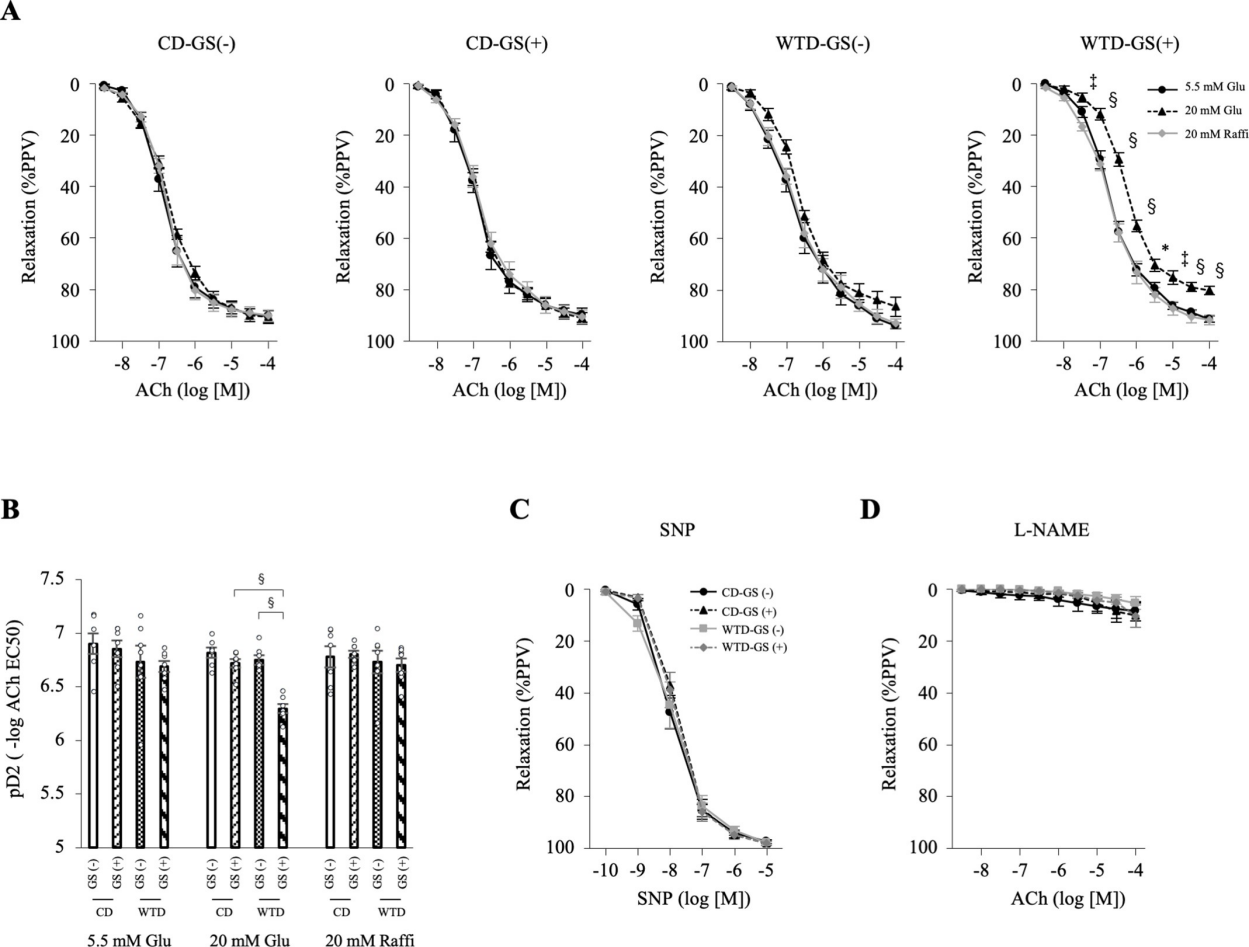
GSs deteriorate endothelium-dependent relaxation in diet-induced obese rats. A: Curves of EDR in the thoracic aorta in response to ACh under 5.5 mM glucose, 20 mM glucose and 20 mM raffinose conditions. * *P* < 0.05, ‡ *P* < 0.005, § *P* < 0.001 compared with 5.5 mM glucose and 20 mM raffinose in the WTD-GS (+) group, one-way repeated-measures ANOVA with the Bonferroni post hoc test. B: Vascular sensitivity, plotted as pD2 (- log of the half-maximal effective concentration [EC50]) of ACh. A significant interaction was detected between diet and GS factors under 20 mM glucose conditions. §*P* < 0.001, two-way ANOVA followed by a simple-effects analysis. C: Curves of vasorelaxation in response to SNP under 20 mM glucose conditions (7 rats per group). D: Curves of EDR in response to ACh in the presence of L-NAME and 20 mM glucose, two-way repeated-measures ANOVA. The data are presented as the means ± SEM. N = 7 rats per group. Glu, glucose; Raffi, raffinose; ACh, acetylcholine; SNP, nitroprusside; L-NAME, an NO synthase inhibitor; CD, control diet; WTD, Western-type diet; GS, glucose spike.

We compared endothelial function among four groups to investigate the difference in EDR between the CD group (nonobese rats) and WTD group (obese rats) with or without GSs: CD-GS (-), CD-GS (+), WTD-GS (-) and WTD-GS (+). The EDR of the thoracic aortas from the CD-GS (-), CD-GS (+) and WTD-GS (-) groups did not change under normal (5.5 mM) or high-glucose (20 mM) conditions, but the EDR of those from the WTD-GS (+) group deteriorated under high-glucose conditions (Fig 2A). This deterioration of EDR was not reproduced in the presence of 20 mM raffinose, an osmotic control for 20 mM glucose (Fig 2A). Among the four groups treated with 20 mM glucose, a significant interaction effect of diet and GSs on pD2 (-log ACh EC 50) was detected (F [1, 24] = 17.4, *P* < 0.001). The simple-effects analysis revealed that the WTD or GS (+) alone did not cause endothelial dysfunction; however, the combination of the WTD and GS (+) deteriorated endothelial function (Fig 2B). No significant differences in the dose-response curves to SNP, an endothelium-independent vasodilator, or ACh were observed in the presence of L-NAME, a NOS inhibitor, even in animals treated with 20 mM glucose, among the four groups (Fig 2C and 2D), suggesting that the impaired endothelial function in the WTD-GS (+) group treated with 20 mM glucose was caused by a deterioration of NO-dependent relaxation. These results indicate that short-term repeated GSs deteriorated EDR only in diet-induced obese rats under high-glucose conditions but not in nonobese rats.

### Endothelial dysfunction caused by repeated GSs in Diet-induced obese rats is independent of fat mass and blood lipid profiles

**Fig 3:**
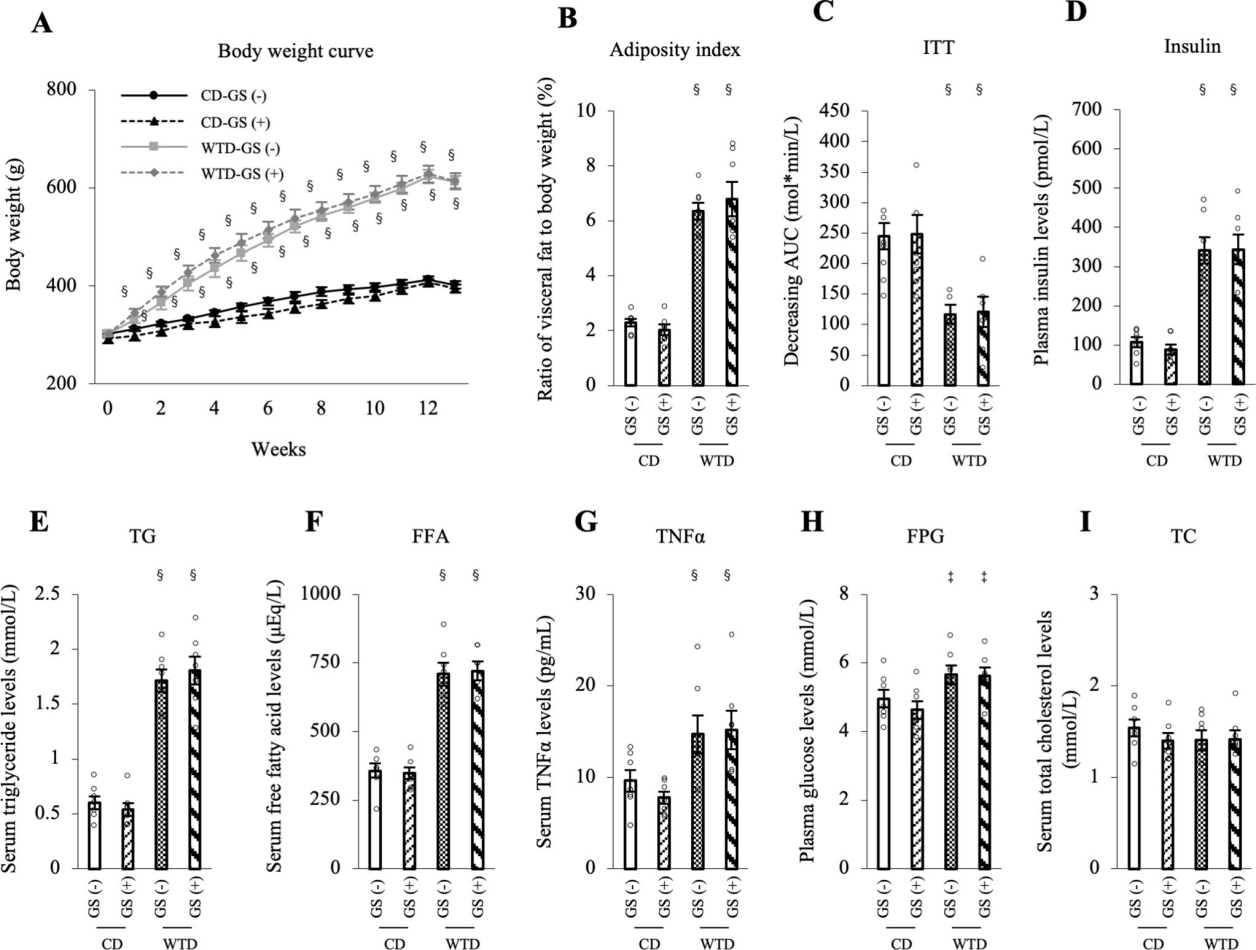
Endothelial dysfunction in the WTD-GS (+) group is independent of metabolic parameters. A: Body weight curve over the duration of feeding and administration. § *P* < 0.001 comparison between diet groups, two-way repeated-measures ANOVA. B: Adiposity index (the ratio of epididymal, retroperitoneal and mesenteric fat mass to body weight). C: ITT. Insulin resistance is presented as the decreasing glucose AUC. D-I: Plasma insulin (D), serum TG (E), FFA (F), TNFα (G), FPG (H), and serum TC (I) levels. ‡ *P* < 0.005 and § *P* < 0.001 for the comparison between diet groups, two-way ANOVA. No interaction was detected between diet and GS factors. Data are presented as the means ± SEM. N = 7 rats per group. CD, control diet; WTD, Western-type diet; GS, glucose spike.

Metabolic parameters were evaluated to understand the mechanism of endothelial dysfunction in the WTD-GS (+) group. A significant main effect of diet was observed in some dependent variables as below without diet/GS interaction, although the main effect of GSs was not significant; body weight gain and visceral fat (adiposity index) were significantly higher (Fig 3A and 3B), the ITT (decreasing AUC) was lower (Fig 3C), and plasma insulin, serum TG, FFA, TNFα, FPG levels and HOMA-IR in the WTD group were higher than those in the CD group (Figs 3D–3H and S2A). Total cholesterol levels were comparable between the two groups (Fig 3I). Systolic and diastolic blood pressure in the WTD group tended to be higher than those in the CD group (the main effect of diet; F [1, 24] = 3.93, *P* = 0.059; F [1, 24] = 3.14, *P* = 0.089, respectively), but the differences were not significant (S2B and S2C Fig). These results indicate that changes in these parameters in the WTD group are consistent with metabolic syndrome. However, GSs did not affect these metabolic parameters in either the CD or WTD group.

Furthermore, the correlation between each metabolic parameter and endothelial function, represented as pD2 (-log ACh EC 50), was assessed to clarify whether the endothelial dysfunction observed in the WTD-GS (+) group under the 20 mM glucose condition was associated with these metabolic parameters. Among these parameters, HOMA-IR and serum FFA and TNFα levels were negatively correlated, and ITT (decreasing AUC) was positively correlated with the pD2 of the WTD-GS (+) group under the 20 mM glucose condition (S3D, S3E, S3G and S3H Fig). Other metabolic parameters, such as body weight, adiposity index, FPG and serum TG levels, were not significantly correlated with pD2 (S3A–S3C and S3F Fig). Based on these results, insulin resistance is important for GS-induced endothelial dysfunction, and metabolic syndrome-related factors such as FFAs and TNFα render endothelial function vulnerable to repeated GSs.

### A NOX inhibitor, SOD and catalase ameliorate endothelial dysfunction caused by high-glucose conditions in diet-induced obese rats with repeated GSs

**Fig 4:**
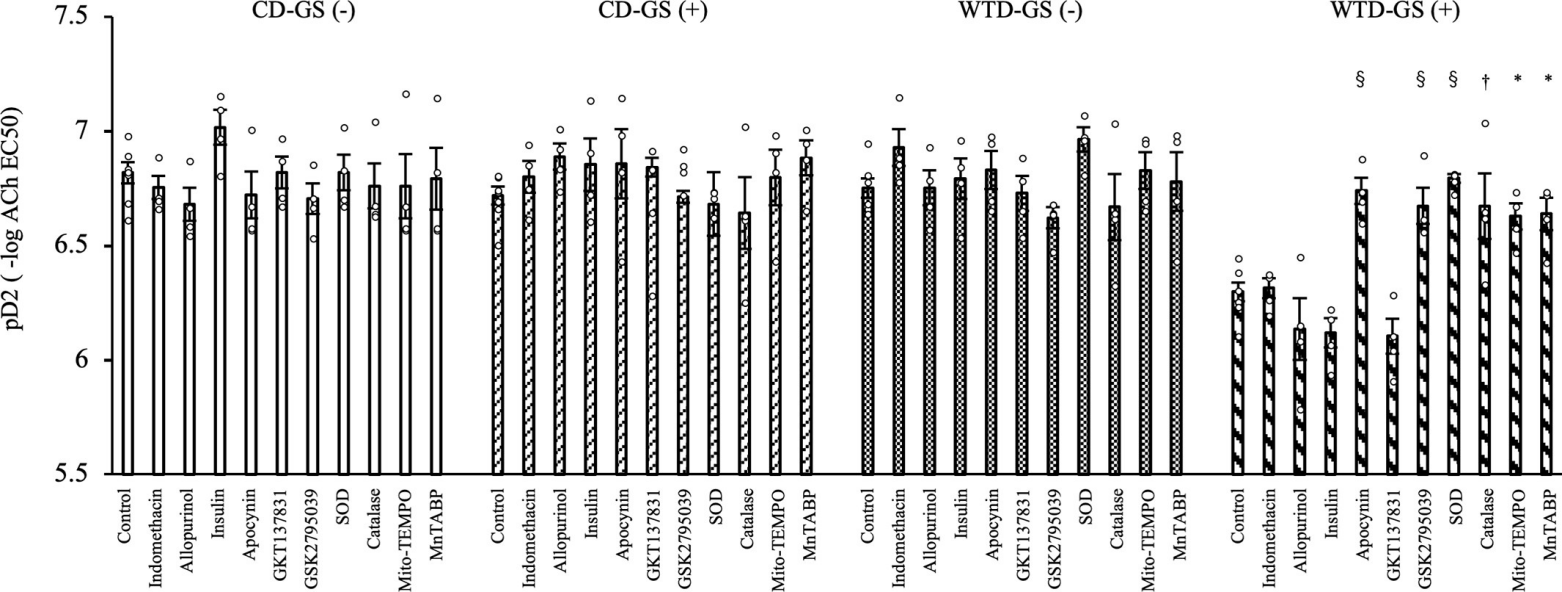
NOX inhibitors, SOD and catalase ameliorate endothelial dysfunction in the WTD-GS (+) group. Vascular sensitivity measured under 20 mM glucose conditions was plotted as pD2 (- log of the half-maximal effective concentration [EC50]) of ACh. The control was administered to 7 rats, and other agents were administered to 4 rats each. * *P* < 0.05, † *P* < 0.01, and § *P* < 0.001 compared with the control in the WTD-GS (+) group, Dunnett’s test. The data are presented as the means ± SEM. Indomethacin, a cyclooxygenase inhibitor; allopurinol, a xanthine oxidase inhibitor; apocynin, a NADPH oxidase (NOX) inhibitor; GKT137831, a NOX1 and 4 inhibitor; GSK2795039, a NOX2 inhibitor; SOD, superoxide dismutase; Mito-TEMPO, a mitochondria-targeted superoxide scavenger; MnTABP, a superoxide dismutase mimetic and peroxynitrite selective scavenger; CD, control diet; WTD, Western-type diet; GS, glucose spike.

We used several pharmacological agents to evaluate endothelial function under 20 mM glucose conditions and to clarify the mechanism of endothelial dysfunction in the WTD-GS (+) group. Apocynin, a NOX inhibitor, improved EDR, which is presented as pD2, in the WTD-GS (+) group without changes in response to SNP (Fig 4; whole response curves to ACh and SNP are shown in S4D and S5D Figs, respectively) (*P* < 0.001). Although GKT137831, a NOX 1 and 4 inhibitor, did not improve EDR, GSK2795039, a NOX 2 inhibitor, ameliorated endothelial dysfunction in the WTD-GS (+) group (Figs 4, S4E, S4F, S5E and S5F) (*P* < 0.001), suggesting that NOX2 was involved in endothelial dysfunction in this group. Similarly, extrinsic SOD and catalase, which are superoxide scavengers and hydrogen peroxide scavengers, also improved EDR (Figs 4, S4G, S4H, S5G and S5H) (*P* < 0.001 and *P* = 0.005, respectively). We investigated this finding in more detail using Mito-TEMPO and MnTABP, a mitochondria-targeted superoxide scavenger and a peroxynitrite selective scavenger, respectively. Both of these agents also improved EDR in the WTD-GS (+) group (Figs 4, S4I, S4J, S5I and S5J) (*P* = 0.017 and *P* = 0.013, respectively), suggesting that mitochondria-derived ROS were also involved in endothelial dysfunction in this group. Indomethacin and allopurinol, a cyclooxygenase inhibitor and a xanthine oxidase inhibitor, respectively, did not improve EDR in the WTD-GS (+) group (Figs 4, S4A, S4B, S5A and S5B), suggesting that prostaglandins and xanthine oxidase-derived radicals are unlikely to be responsible for endothelial dysfunction. We also evaluated the effect of insulin on endothelial function because repeated GSs were accompanied by transient hyperinsulinemia, and we found that even a high dose of insulin (10 nM) did not affect EDR (Figs 4, S4C and S5C), suggesting that direct exposure of the endothelium to insulin is neither harmful nor protective.

### Expression of the NOX2 mRNA Is upregulated and SOD2 and catalase mRNAs are downregulated in thoracic aortas from diet-induced obese rats with repeated GSs

**Fig 5:**
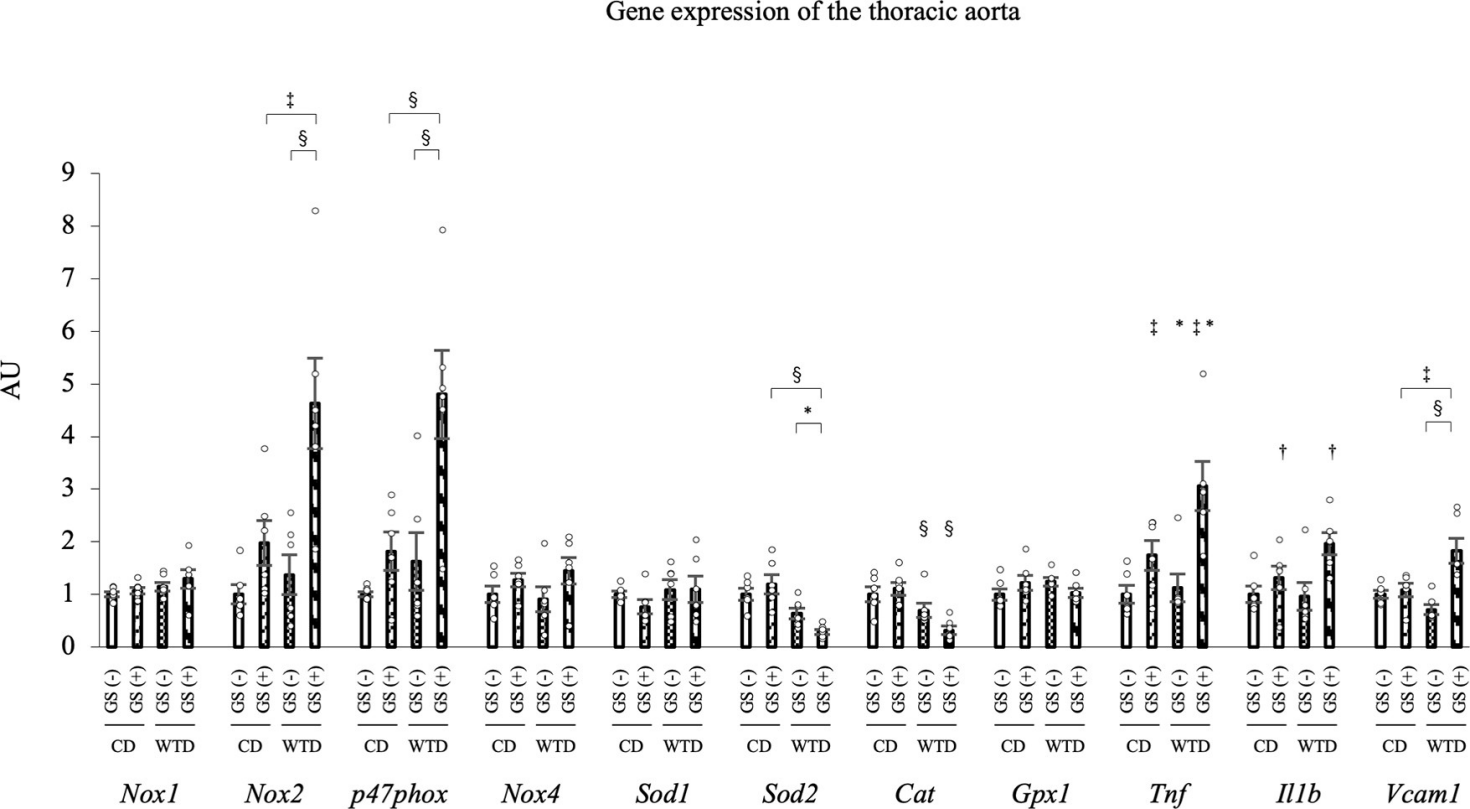
Quantitative PCR analysis of mRNA expression in the thoracic aorta. Expression of the NOX2 and SOD2 mRNAs was synergistically altered by the combination of a WTD and repeated GSs. The significant interactive effect of diet and GS on the mRNA expression of NOX2, p47phox, SOD2 and VCAM1 was confirmed by two-way ANOVA followed by a simple-effects analysis (* *P* < 0.05, ‡ *P* < 0.005, § *P* < 0.001). Catalase mRNA expression was significantly different between groups with different diets, TNFα mRNA expression was different between groups with different diet or GS factors and IL1β mRNA expression was different between groups with different GS factors without a diet/GS interaction. * *P* < 0.05, † *P* < 0.01, and ‡ *P* < 0.005 for the comparison between diet or GS factors, two-way ANOVA. The data are presented as the means ± SEM. N = 6 rats per group. CD, control diet; WTD, Western-type diet; GS, glucose spike.

Then, we analyzed the expression of the oxidoreductase genes in the thoracic aortas. Among the four groups, the main effect of diet on the expression of the NOX2 and p47phox mRNAs was significant (F [1, 20] = 8.26, *P* = 0.009; F [1, 20] = 11.45, *P* = 0.003, respectively). The main effect of GS on NOX2 and p47phox expression was also significant (F [1, 20] = 16.21, *P* < 0.001; F [1, 20] = 14.07, *P* = 0.001, respectively). However, these main effects were qualified by a significant interaction between diet and GS (F [1, 20] = 4.69, *P* = 0.043; F [1, 20] = 4.90, *P* = 0.039, respectively). Similarly, the main effect of diet on SOD2 expression was significant (F [1, 20] = 28.39, *P* < 0.001), but this main effect was qualified by a significant interaction between diet and GS (F [1, 20] = 5.20, *P* = 0.034). The simple-effects analysis revealed that the combination of a WTD and GS (+) upregulated the expression of the NOX2 and p47phox mRNAs and downregulated SOD2 mRNA expression. The main effect of diet on the expression of the catalase mRNA was confirmed (F [1, 20] = 19.85, *P* < 0.001) without a diet/GS interaction (F [1, 20] = 3.94, *P* = 0.061) (Fig 5). Notably, no significant differences in the mRNA expression of other redox-related enzymes, such as NOX1, NOX4, SOD1 and GPX1, were observed among all four groups (Fig 5). Based on these results, 1) WTD-induced obesity downregulates the catalase mRNA; and 2) the combination of repeated GSs and diet-induced obesity synergistically upregulates the NOX2 and p47phox mRNAs and downregulates the SOD2 mRNA. Regarding inflammatory genes, TNFα mRNA expression was significantly different between groups with different diet or GS factors (F [1, 20] = 5.26, *P* = 0.033; F [1, 20] = 17.99, *P* < 0.001, respectively) and IL1β mRNA expression was also different between groups with different GS factors (F [1, 20] = 9.17, *P* = 0.007) without a diet/GS interaction (Fig 5). An interactive effect of diet and GS on the expression of the VCAM1 mRNA was detected (F [1, 20] = 12.0, *P* = 0.002), and the simple-effects analysis revealed that the combination of a WTD and GS (+) upregulated the VCAM1 mRNA (Fig 5).

### Free radical formation is increased by repeated GSs in the thoracic aortas of diet-induced obese rats

**Fig 6:**
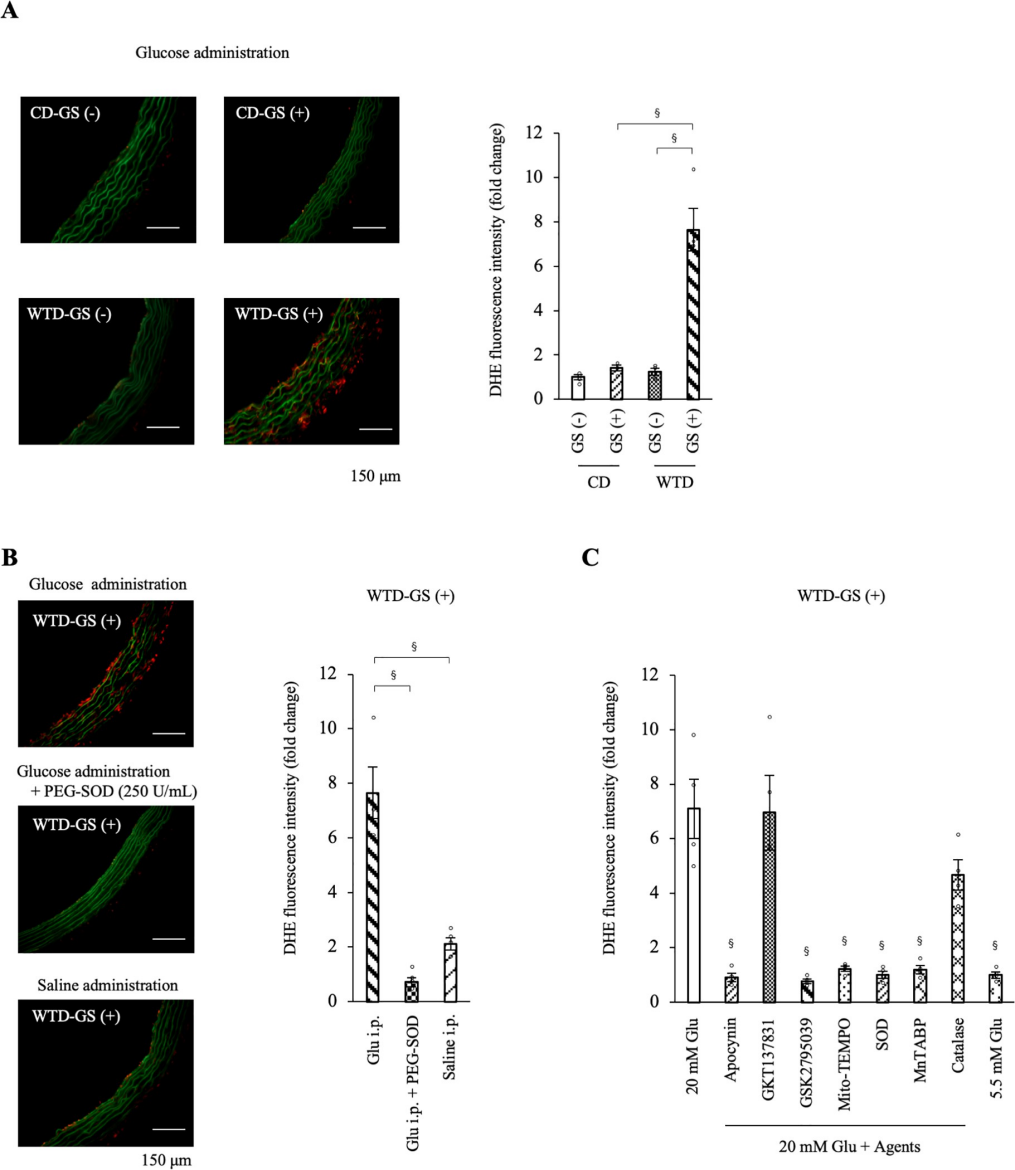
ROS production is increased by glucose spikes only in the WTD-GS (+) group and diminished by NOX2 inhibitor and a mitochondria-targeted superoxide scavenger. A: Representative images of DHE staining and the DHE fluorescence intensity in the thoracic aorta removed 2 hours after the intraperitoneal administration of glucose (1 g/kg). The significant interactive effect of diet and GS on DHE fluorescence intensity was confirmed by two-way ANOVA followed by a simple-effects analysis (§ *P* < 0.001). B: DHE fluorescence intensity in the aortas incubated with PEG-SOD and in the aortas removed after the intraperitoneal administration of saline to the WTD-GS (+) group. The control group (Glu i.p.) in the graph was the same as the WTD-GS (+) group in Fig 4A. § *P* < 0.001, one-way ANOVA. C: DHE fluorescence intensity in the aortas removed from the WTD-GS (+) group after equilibration in 20 mM glucose for 2 hours and with several pharmacological agents for 30 minutes. The 5.5 mM Glu group was composed of aortas removed from the WTD-GS (+) group after equilibration in 5.5 mM glucose for 2 hours without any pharmacological agents. § *P* < 0.001 compared with the 20 mM Glu group, Dunnett’s test. The DHE fluorescence intensity is presented as fold changes in fluorescence intensity relative to that of the 5.5 mM Glu group. The data are presented as the means ± SEM. N = 4 rats per group. Glu, glucose; i.p., intraperitoneal; PEG-SOD, polyethylene glycol-superoxide dismutase; apocynin, an NADPH oxidase (NOX) inhibitor; GKT137831, a NOX1 and 4 inhibitor; GSK2795039, a NOX2 inhibitor; Mito-TEMPO, a mitochondria-targeted superoxide scavenger; SOD, superoxide dismutase; MnTABP, a superoxide dismutase mimetic and peroxynitrite selective scavenger; CD, control diet; WTD, Western-type diet; GS, glucose spike.

Repeated GSs in diet-induced obese rats resulted in an imbalance in redox enzyme mRNA expression. ROS levels were evaluated by performing DHE staining of thoracic aortas isolated 2 hours after the administration of glucose (1 g/kg BW) or saline to investigate free radical formation in the aorta. The fluorescence intensity of DHE was higher in the WTD-GS (+) group administered glucose than in the other three groups (Fig 6A). Among the four groups, the interactive effect of diet and GS on the fluorescence intensity of DHE (F [1, 12] = 37.42, *P* < 0.001) was confirmed by two-way ANOVA (Fig 6A). Simple-effects analysis revealed that the combination of a WTD and GS (+) increased the fluorescence intensity of DHE, although a WTD or GS (+) alone did not. This increased intensity of DHE in the WTD-GS (+) group was diminished by a preincubation with 250 U/mL PEG-SOD (Fig 6B) (*P* < 0.001). In addition, the WTD-GS (+) group administered saline showed a significantly lower fluorescence intensity of DHE than the WTD-GS (+) group administered glucose (Fig 6B) (*P* < 0.001). These results were consistent with those of the vascular reactivity experiment in which endothelial function deteriorated only under high-glucose conditions in the WTD-GS (+) group.

Furthermore, we evaluated the ROS source using thoracic aortas isolated from WTD-GS (+) groups after equilibration in 20 mM glucose for 2 hours in the presence of several pharmacological agents for 30 minutes. We confirmed a higher fluorescence intensity of DHE in aortas after exposure to 20 mM glucose than after exposure to 5.5 mM glucose (Fig 6C; representative images of DHE staining are shown in S6A and S6I Fig) (*P* < 0.001). This increased intensity was significantly diminished by apocynin, GSK2795039 (a NOX2 inhibitor) and Mito-TEMPO (a mitochondria-targeted superoxide scavenger) (*P* < 0.001) but not diminished by GKT137831 (a NOX1 and 4 inhibitor) (Figs 6C and S6B–S6E) (*P* = 0.942). In addition, SOD and MnTABP, a superoxide dismutase mimetic and peroxynitrite selective scavenger, also significantly reduced this increased intensity of DHE (Figs 6C, S6F and S6G) (*P* < 0.001). On the other hand, catalase only slightly but not significantly diminished the DHE intensity (Figs 6C and S6H) (*P* = 0.056). These results suggest that the ROS detected using DHE staining were mainly composed of superoxide anions, perhaps partially including hydrogen peroxide, and the ROS source was derived from NOX2 and mitochondria in the WTD-GS (+) group under high-glucose conditions.

### CDDO-Me protects the endothelial function of diet-induced obese rats against repeated GSs

Because repeated GSs induced oxidative stress in the thoracic aortas of diet-induced obese rats, we evaluated whether the administration of CDDO-Me, an activator of the Nrf2 system, prevented endothelial dysfunction. We determined the optimal dose of CDDO-Me by orally administering five doses of CDDO-Me (0 [vehicle], 0.3, 1, 3 and 15 mg/kg BW) to rats in the WTD-GS (+) group. As shown in S7A and S7B Fig, endothelial function was most strongly preserved after exposure to repeated GSs and 3 mg/kg BW, although this protective effect was diminished in animals treated with 15 mg/kg BW CDDO-Me.

**Fig 7:**
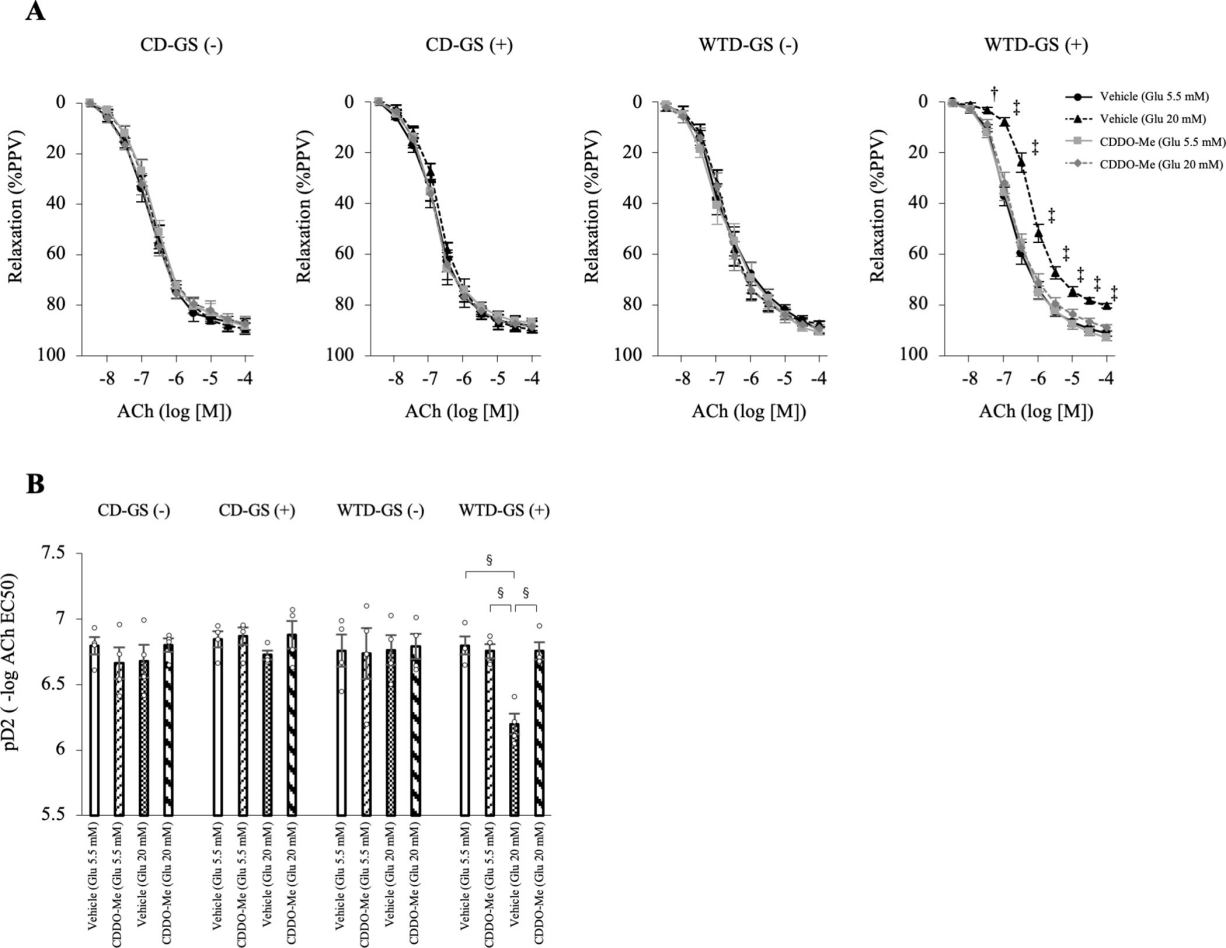
CDDO-Me (3 mg/kg) protects endothelial function in diet-induced obese rats against repeated glucose spikes. Rats were fed a CD or WTD for 13 weeks, treated with vehicle or CDDO-Me for 2 weeks and administered saline or glucose for 1 week. A: Curves of endothelium-dependent relaxation in response to ACh under 5.5 mM glucose or 20 mM glucose conditions. † *P* < 0.01 and ‡ *P* < 0.005 compared with the other three groups, one-way repeated-measures ANOVA with the Bonferroni post hoc test. B: Vascular sensitivity, plotted as pD2 (- log of the half-maximal effective concentration [EC50]) of ACh. § *P* < 0.001, one-way ANOVA with the Bonferroni post hoc test. The data are presented as the means ± SEM. N = 4 rats per group. Glu, glucose; ACh, acetylcholine; CD, control diet; WTD, Western-type diet; GS, glucose spike.

We orally administered vehicle (sesame oil) or CDDO-Me (3 mg/kg BW) to four groups of 19-week-old rats for two weeks and intraperitoneally administered saline or glucose for one week beginning at 20 weeks old, similar to the first cohort. Thoracic aortas were isolated from these groups, and EDR was evaluated after exposure to 5.5 mM or 20 mM glucose. After treatment with 20 mM glucose, the administration of CDDO-Me to rats in the WTD-GS (+) group significantly ameliorated EDR and pD2 compared with vehicle (*P* < 0.001), although no significant differences were observed in animals treated with 5.5 mM glucose (Fig 7A and 7B). Among the CD-GS (-), CD-GS (+), and WTD-GS (-) groups, no differences in EDR or pD2 were observed among the vehicle and CDDO-Me groups after treatment with either 5.5 mM or 20 mM glucose (Fig 7A and 7B).

### CDDO-Me does not affect metabolic parameters

**Fig 8:**
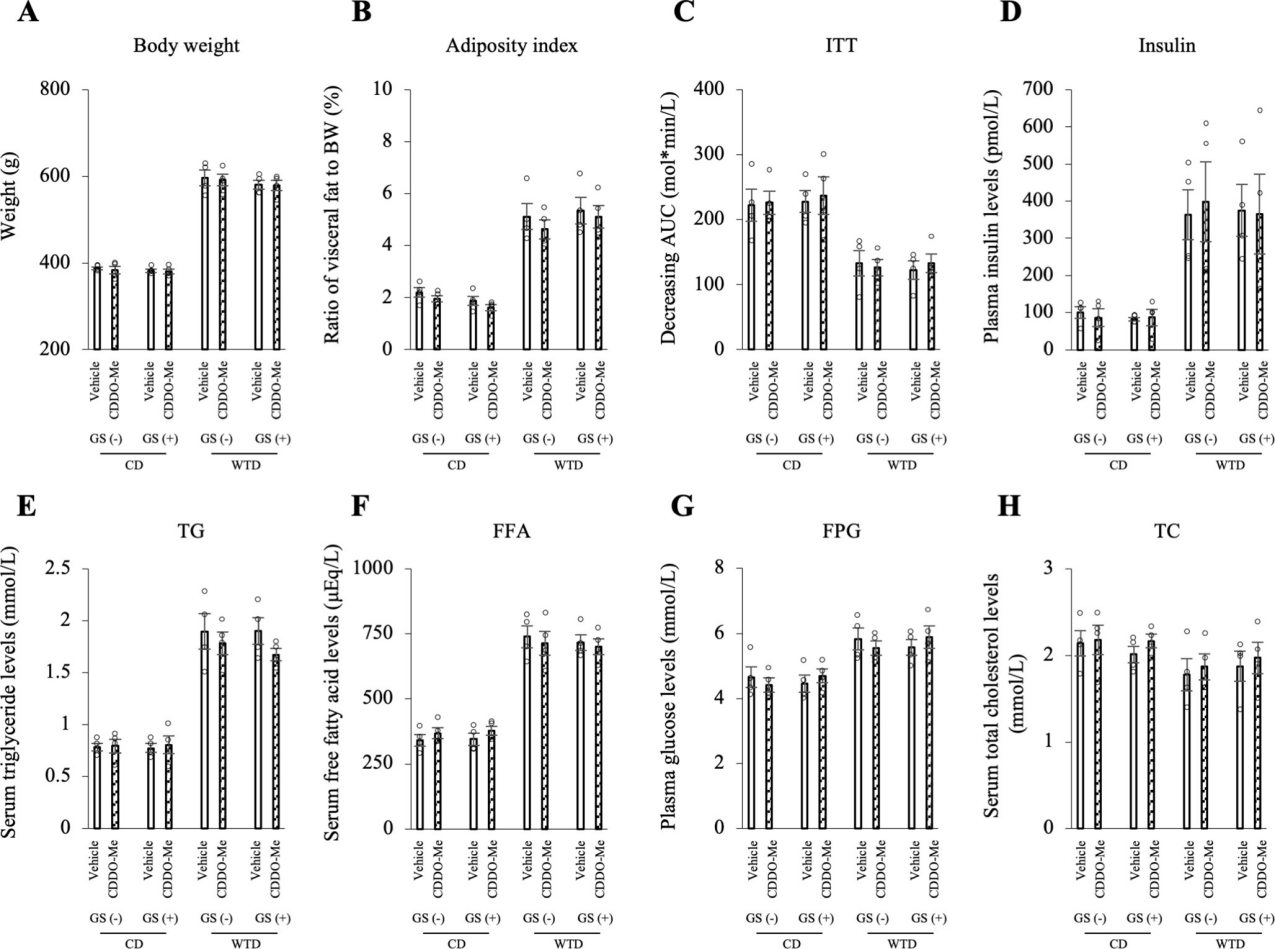
CDDO-Me (3 mg/kg) does not affect the body weight, fat mass or blood lipid profile of any group. A: Body weight over the duration of feeding and administration. B: Adiposity index (the ratio of epididymal, retroperitoneal and mesenteric fat mass to body weight). C: ITT. Insulin resistance is presented as the decreasing glucose AUC. D-H: Plasma insulin (D), serum TG (E), FFA (F), FPG (G) and serum TC (H) levels. No significant differences were observed between the vehicle and CDDO-Me in each group using the Bonferroni correction. The data are presented as the means ± SEM. N = 4 rats per group. CD, control diet; WTD, Western-type diet; GS, glucose spike.

The effects of CDDO-Me on metabolic parameters were assessed. In the comparison of treatment with vehicle or CDDO-Me for each group, no significant differences in body weight, adiposity index (visceral fat mass), ITT (insulin resistance), or plasma insulin, serum TG, FFA, FPG, or serum TC levels were observed (Fig 8A–8H). Additionally, no differences in HOMA-IR, serum TNFα levels or blood pressure were observed (S8A–S8D Fig).

### CDDO-Me suppresses NOX2 mRNA expression and Increases SOD2 and catalase mRNA levels in the thoracic aortas of diet-induced obese rats with repeated GSs

**Fig 9:**
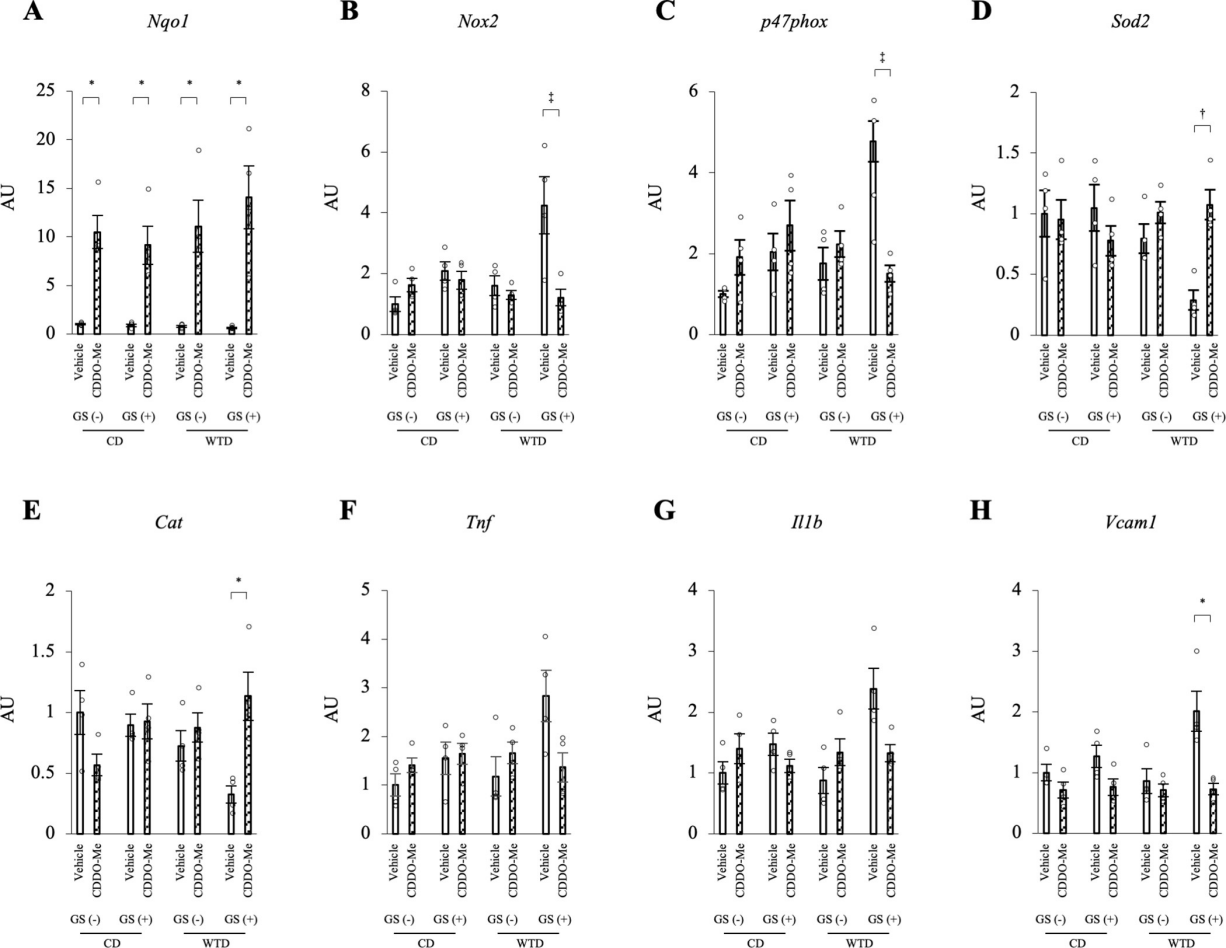
The effect of CDDO-Me (3 mg/kg) on gene expression. CDDO-Me suppresses the expression of the NOX2 mRNA and increases SOD2 and catalase mRNA expression in the thoracic aorta of the WTD-GS (+) group. A-H: Quantitative PCR analysis of the expression of NQO1 (A), NOX2 (B), p47phox (C), SOD2 (D), catalase (E), TNFα (F), IL1β (G) and VCAM1 (H) mRNAs in the thoracic aorta. * *P* < 0.05, † *P* < 0.01, and ‡ *P* < 0.005, Bonferroni correction. The data are presented as the means ± SEM. N = 4 rats per group. CD, control diet; WTD, Western-type diet; GS, glucose spike.

We evaluated the changes in gene expression in the thoracic aortas between the vehicle and CDDO-Me groups to clarify the mechanism by which CDDO-Me ameliorated endothelial dysfunction. The mRNA expression of NQO1, HMOX1 and GSTP1, a target gene of the Nrf2 system, was markedly increased by CDDO-Me in the four groups (Figs 9A, S9A and S9B). CDDO-Me significantly reduced the expression of the NOX2 and p47phox mRNAs (*P* = 0.004 and *P* = 0.004, respectively) and significantly increased SOD2 and catalase mRNA levels (*P* = 0.007 and *P* = 0.033, respectively) in the WTD-GS (+) group (Fig 9B–9E). Regarding inflammatory genes, the VCAM1 mRNA was expressed at significantly lower levels in the WTD-GS (+) group treated with CDDO-Me than in the WTD-GS (+) group treated with vehicle (Fig 9H) (*P* = 0.039). CDDO-Me also reduced levels of the TNFα and IL1β mRNAs, but the differences between vehicle and CDDO-Me treatments were not significant (Fig 9F and 9G).

### CDDO-Me suppresses local and systemic oxidative stress caused by repeated GSs in diet-induced obese rats

**Fig 10:**
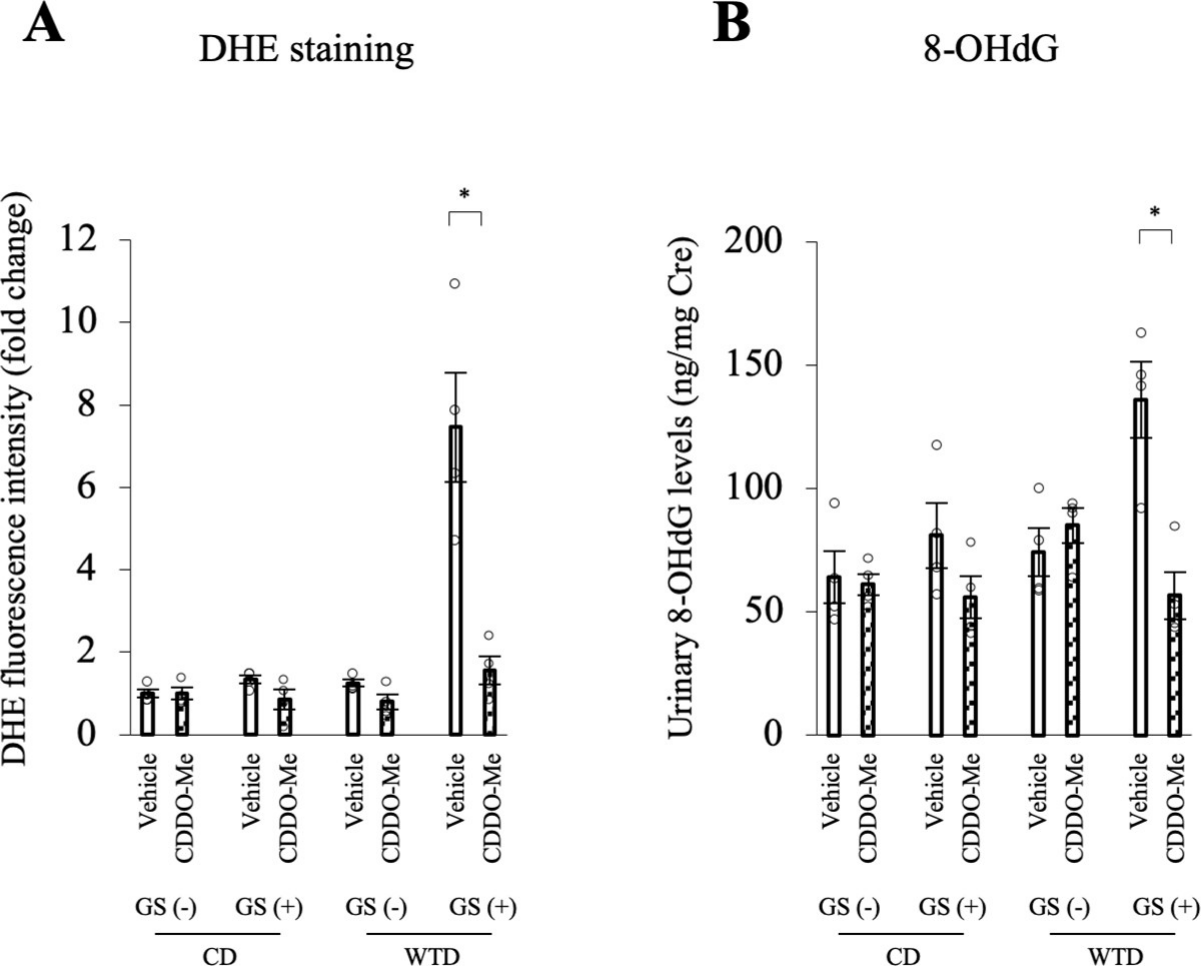
CDDO-Me (3 mg/kg) suppresses local and systemic oxidative stress in the WTD-GS (+) group. The thoracic aorta and urinary sample were collected 2 hours after glucose administration. A: DHE fluorescence intensity in the thoracic aorta. B: Urinary 8-OHdG levels. * *P* < 0.05, Bonferroni correction. The data are presented as the means ± SEM. N = 4 rats per group. CD, control diet; WTD, Western-type diet; GS, glucose spike.

We measured the DHE fluorescence intensity in the thoracic aortas and urinary 8-OHdG levels in the vehicle and CDDO-Me groups to evaluate the effect of CDDO-Me on local and systemic oxidative stress. These samples were collected 2 hours after the intraperitoneal administration of glucose (1 g/kg BW). Treatment with CDDO-Me in the WTD-GS (+) group significantly decreased the DHE fluorescence intensity and urinary 8-OHdG levels (Fig 10A and 10B; representative images of DHE staining are shown in S10 Fig) (*P* = 0.020 and *P* = 0.048, respectively). In the other three groups, no significant differences in the DHE fluorescence intensity or urinary 8-OHdG levels were observed between the vehicle and CDDO-Me groups (Figs 10A, 10B and S10).

## Discussion

In this study, we investigated the individual and combined effects of insulin resistance and repeated GSs on the endothelial function of the murine thoracic aorta. We noted that the combination of three factors, i.e., diet-induced insulin resistance, short-term repeated GSs, and transient exposure to high-glucose conditions ex vivo, was necessary to cause endothelial dysfunction.

When incubated with high glucose, only aortic rings isolated from the WTD-GS (+) groups exhibited a significant impairment in endothelial function, suggesting that repeated GSs in an insulin resistance state alter the endothelial-dependent vascular reactivity to high glucose. However, our findings demand a rational explanation for why neither diet-induced insulin resistance nor repeated GSs alone rendered the endothelial function of our murine model susceptible to a transient hyperglycemic state. Consistent with previous reports [34,35], at the molecular level, diet-induced obesity caused a decrease in antioxidant enzyme (SOD2 and catalase) mRNA expression without any change in pro-oxidant (NOX2) mRNA expression in murine aortas. On the other hand, and consistent with a previous report [36], pure glycemic spikes caused an increase in NOX2 mRNA expression. These changes were, however, inadequate to render aortic endothelial function vulnerable to high-glucose conditions. Nevertheless, a combination of diet-induced obesity and repeated GSs synergistically enhanced the imbalanced mRNA expression of these redox enzymes in the aorta, thus resulting in one plausible explanation for the observed phenomenon. Although the mechanism underlying the synergistic effect of diet-induced obesity and repeated GSs on the observed imbalance in redox gene expression in the aorta is unclear, hyper-free fatty acidemia associated with obesity might contribute. Increased serum FFA levels accompanying obesity have been reported to cause an increase in NOX mRNA expression and a decrease in the expression of genes encoding antioxidant enzymes (NQO1, SOD and catalase) in endothelial cells [37,38]. In our experiment, serum FFA levels correlated negatively with pD2 under high-glucose conditions (S3G Fig), further suggesting that repeated GSs and hyper-free fatty acidemia accompanying obesity may have acted synergistically to cause an imbalance in redox enzyme expression in the aorta of our murine model.

Another phenomenon requiring an explanation is the impaired endothelial function of aortic rings isolated from the WTD-GS (+) groups following exposure to high-glucose conditions ex vivo. Studies have established that endothelial cells tend to increase glucose uptake in an insulin-independent manner under high-glucose conditions, mainly through glucose transporter 1 (GLUT1), whose expression has never been shown to be affected by high glucose [39]. This increased uptake has been implicated in increasing the generation of mitochondrial-derived ROS [40–44]. This result is again consistent with our experiment where endothelial dysfunction and increased ROS production were only observed under high-glucose conditions. The ability of a mitochondrial-targeted superoxide scavenger (Mito-TEMPO) to suppress ROS generation and improve endothelial dysfunction induced by high glucose in aortic rings isolated from the WTD-GS (+) group further supports the hypothesis that increased mitochondrial-derived ROS generation was responsible for high glucose-induced endothelial dysfunction in aortic rings isolated from this experimental group. However, why was this phenomenon not observed when aortic rings from other experimental groups were exposed to high-glucose conditions? As mentioned above, significant disequilibrium of redox gene expression induced by the combination of insulin resistance and repeated GSs in this group provides a plausible explanation. SOD2, which is localized in the mitochondria and eliminates mitochondria-derived ROS [45], was downregulated in the WTD-GS (+) group; again, NOX2, which has also been implicated in increased mitochondrial-derived ROS under high-glucose conditions [46], was upregulated in the WTD-GS (+) groups. The ability of GSK2795039 (a pharmacological inhibitor of NOX2) and SOD to improve high glucose-induced endothelial dysfunction and attenuate ROS production in the WTD-GS (+) groups in the current experiment further confirms the previously established findings. In summary, the redox imbalance in aortas from the WTD-GS (+) group rendered endothelial function vulnerable to high-glucose conditions.

In this experiment, a nonspecific measure of ROS production (DHE staining) was employed. Since this method cannot identify the specific type of ROS, hydrogen peroxide rather than superoxide radicals alone [47] may have contributed to the observed endothelial dysfunction in the WTD-GS (+) group. However, the effect of catalase on reducing the DHE fluorescence intensity was limited, suggesting that the ROS detected based on DHE fluorescence were mainly composed of superoxide. Because decreased NO bioavailability and subsequent endothelial dysfunction are generally caused by superoxide [48–51], the observed endothelial dysfunction in our experiment was more likely caused by high glucose-induced mitochondrial-derived superoxide generation. Nevertheless, since SOD catalyzes the dismutation of superoxide, generating hydrogen peroxide as a byproduct, quantitation of hydrogen production will be needed to ascertain its contribution to endothelial dysfunction [52].

In contrast to previous reports, neither pure GSs nor diet-induced obesity alone caused endothelial dysfunction in the current study. According to a previous report [9], at least 3 months of repeated GSs were necessary to enhance atherogenesis. The period of GSs in our experiment may thus be too short to induce endothelial dysfunction. On the other hand, although high-fat diet (HFD)-induced obesity has generally been reported to cause endothelial dysfunction [53], the composition of HFDs, particularly that of saturated fatty acids (palmitic and stearic acids), is an important determinant. For example, feeding rats a 49.2% HFD composed of 80% nonsaturated fatty acids for 27 weeks did not impair endothelial function even in obesity [54]. Similarly, a 45% HFD composed of 68.6% nonsaturated fatty acids or 59% HFD composed of 59% nonsaturated fatty acids did not cause endothelial dysfunction [55,56]. Even a 58% HFD composed of 93.3% saturated fatty acids derived from coconut oil did not cause endothelial dysfunction [57] because it contained a large amount of lauric acid (47.5%) and a small amount of palmitic acid (9.5%) and stearic acid (10.9%). The WTD used in our experiment contains a low fat content (39.9%). Moreover, the overall composition of saturated fatty acids (palmitic and stearic acid) was even lower, 1.6% and 9.7%, respectively. In addition, we performed ex vivo evaluation of the endothelial dysfunction, which is generally less sensitive than the in vivo approach because the pro-inflammatory perivascular adipose tissue (PVAT) is removed while conducting wire myograph experiments. This approach might have prevented us from detecting the expected HFD-induced endothelial dysfunction. Nevertheless, evidence shows that the thoracic aorta is more resistant to HFD-induced effects than other vessels even in vivo because of its characteristic PVAT related to brown-like adipose tissue. Actually, the magnetic resonance imaging (MRI)-based method, which can evaluate the endothelial function in vivo, revealed that short-term feeding with an HFD induced the endothelial dysfunction in the abdominal aorta and coronary microcirculation for only 2 weeks or 7 days, respectively, but not in the thoracic aorta even for 8 weeks [58,59]. These findings may explain why WTD feeding on its own did not cause endothelial dysfunction in our experiment. However, an apparent strength of this experiment is the fact that although pure GSs and diet-induced obesity were insufficient to cause endothelial dysfunction, we showed that the two factors acted synergistically to render endothelial function vulnerable to high-glucose conditions.

Based on the hypothesis generated in our study that repeated GSs and diet-induced insulin resistance act synergistically to increase the vulnerability of murine aortas to oxidative stress and endothelial dysfunction under high-glucose conditions, we evaluated the effect of CDDO-Me (a Nrf2 activator) on endothelial dysfunction. The administration of CDDO-Me to the WTD-GS (+) group ameliorated endothelial dysfunction, reduced ROS generation, and normalized redox gene expression in the aorta. These findings might be attributed to the ability of CDDO-Me to systematically induce antioxidant enzyme expression, as reported in previous studies [60–63]. CDDO-Me has also been shown to exert an anti-inflammatory effect that is mainly mediated by the inhibition of NF-κB [64]. The ability of CDDO-Me to decrease NOX2 expression, probably due to the suppression of TNFα [65], and increase SOD2 and catalase gene expression in our study further supports the hypothesis that its ability to normalize redox enzyme expression is responsible for the observed attenuation of endothelial dysfunction. These results indicate the possibility of CDDO-Me as a treatment for GS-induced endothelial dysfunction that leads to the development of CVDs.

However, the dose of CDDO-Me may be critical for the treatment of endothelial dysfunction, since a higher concentration of CDDO-Me caused excessive mitochondrial uncoupling [66] and upregulated the expression of the TNFα and monocyte chemotactic protein-1 (MCP-1) mRNAs, resulting in the deterioration of antiatherogenic effects [67]. In the present study, an optimal dosage of CDDO-Me ameliorated endothelial dysfunction and reduced ROS production and proinflammatory gene expression in the aorta of WTD-GS (+) groups, consistent with previous reports [67–69].

In conclusion, for the first time, we have shown that a combination of diet-induced insulin resistance and repeated GSs act synergistically to render the endothelial function of murine aortae vulnerable to high glucose, mainly through an imbalance in redox gene expression. We also showed that the optimal dosage of CDDO-Me corrected this redox imbalance and significantly attenuated endothelial dysfunction.

## Supporting information

S1 Fig Study design. A: First cohort, the comparison of 4 groups: CD-GS (-), CD-GS (+), WTD-GS (-) and WTD-GS (+). Rats were fed a CD or WTD for 13 weeks and administered saline or glucose for 1 week (N = 7 rats per group). Saline and glucose were intraperitoneally administered twice daily for 1 week. B: Second cohort, examination of the effect of CDDO-Me on endothelial function. Rats were fed a CD or WTD for 13 weeks, treated with vehicle or CDDO-Me for 2 weeks and administered saline or glucose for 1 week (N = 4 rats per group). Vehicle (sesame oil) and CDDO-Me were orally administered once daily for 2 weeks. p.o., per os; i.p., intraperitoneal; CD, control diet; WTD, Western-type diet; GS, glucose spike.
(TIFF)

S2 Fig HOMA-IR and blood pressure. A: HOMA-IR. B-C: Systolic and diastolic blood pressure. § *P* < 0.001 for the comparison between diet groups, two-way ANOVA. No interaction was detected between diet and GS factors. Data are presented as the means ± SEM. N = 7 rats per group. HOMA-IR, homeostasis model assessment of insulin resistance; BP, blood pressure; CD, control diet; WTD, Western-type diet; GS, glucose spike.
(TIFF)

S3 Fig Correlation between each parameter and pD2 (- log of the half-maximal effective concentration of ACh). HOMA-IR, serum FFA and TNFα levels were negatively correlated, and the ITT (decreasing AUC) was positively correlated with the pD2 of the WTD-GS (+) group under the 20 mM glucose condition (N = 7). Correlations between body weight (A), adiposity index (B), FPG (C), HOMA-IR (D), ITT (E), serum TG levels (F), serum FFA levels (G) or serum TNFα levels (H) with the pD2 of the WTD-GS (+) group under 20 mM glucose conditions. The adiposity index is the ratio of epididymal, retroperitoneal and mesenteric fat mass to body weight. The ITT is presented as the decreasing glucose AUC. HOMA-IR, homeostasis model assessment of insulin resistance; WTD, Western-type diet; GS, glucose spike; *r*, Pearson’s correlation coefficient.
(TIFF)

S4 Fig Curves of EDR in the thoracic aortas from the WTD-GS (+) group in response to ACh under 20 mM glucose conditions in the presence of several agents. A: Indomethacin, a cyclooxygenase inhibitor; B: allopurinol, a xanthine oxidase inhibitor; C: insulin; D: apocynin, a NADPH oxidase (NOX) inhibitor; E: GKT137831, a NOX1 and 4 inhibitor; F: GSK2795039, a NOX2 inhibitor; G: SOD, superoxide dismutase; H: catalase; I: Mito-TEMPO, a mitochondria-targeted superoxide scavenger; J: MnTABP, a superoxide dismutase mimetic and peroxynitrite selective scavenger. N = 7 (control) or 4 (each agent) rats per group. * *P* < 0.05, † *P* < 0.01, ‡ *P* < 0.005, and § *P* < 0.001 compared with the control, one-way repeated-measures ANOVA. The data are presented as the means ± SEM; WTD, Western-type diet; GS, glucose spike.
(TIFF)

S5 Fig Curves of vasorelaxation in the thoracic aorta from the WTD-GS (+) group in response to SNP under 20 mM glucose conditions in the presence of several agents. None of the agents altered vascular reactivity to SNP. A: Indomethacin, a cyclooxygenase inhibitor; B: allopurinol, a xanthine oxidase inhibitor; C: insulin; D: apocynin, a NADPH oxidase (NOX) inhibitor; E: GKT137831, a NOX1 and 4 inhibitor; F: GSK2795039, a NOX2 inhibitor; G: SOD, superoxide dismutase; H: catalase; I: Mito-TEMPO, a mitochondria-targeted superoxide scavenger; J: MnTABP, a superoxide dismutase mimetic and peroxynitrite selective scavenger. N = 7 (control) or 4 (each agent) rats per group, one-way repeated-measures ANOVA. The data are presented as the means ± SEM; WTD, Western-type diet; GS, glucose spike.
(TIFF)

S6 Fig Representative images of DHE staining in the thoracic aortas removed from the WTD-GS (+) group. A: 20 mM glucose condition. The aortas were equilibrated in 20 mM glucose for 2 hours without any agents. B-H: The aortas were equilibrated in 20 mM glucose for 2 hours in the presence of apocynin (B), GKT137831 (C), GSK2795039 (D), Mito-TEMPO (E), SOD (F), MnTABP (G) and catalase (H) for 30 minutes. I: 5.5 mM glucose condition. The aortas were equilibrated in 5.5 mM glucose for 2 hours with no agents. Apocynin, an NADPH oxidase (NOX) inhibitor; GKT137831, a NOX1 and 4 inhibitor; GSK2795039, a NOX2 inhibitor; Mito-TEMPO, a mitochondria-targeted superoxide scavenger; SOD, superoxide dismutase; MnTABP, a superoxide dismutase mimetic and peroxynitrite selective scavenger.
(TIFF)

S7 Fig CDDO-Me (3 mg/kg) exerts the best vasoprotective effect on the WTD-GS (+) group. A: Curves of EDR in response to ACh under 20 mM glucose conditions after exposure to 5 doses of CDDO-Me (0 [vehicle], 0.3, 1, 3, and 15 mg/kg). * *P* < 0.05, ‡ *P* < 0.005, and § *P* < 0.001 compared with the 0 mg/kg (vehicle) group, one-way repeated-measures ANOVA. B: Vascular sensitivity, plotted as pD2 (- log of the half-maximal effective concentration [EC50]) of ACh. § *P* < 0.001 compared with the 0 mg/kg (vehicle) group, Dunnett’s test. The data are presented as the means ± SEM. N = 4 rats per group. WTD, Western-type diet; GS, glucose spike.
(TIFF)

S8 Fig CDDO-Me (3 mg/kg) did not affect HOMA-IR, serum TNFα levels or blood pressure among all groups. A-D: HOMA-IR (A), serum TNFα levels (B), systolic blood pressure (C) and diastolic blood pressure (D). No significant differences were observed between the vehicle and CDDO-Me in each group using the Bonferroni correction. The data are presented as the means ± SEM. N = 4 rats per group. HOMA-IR, homeostasis model assessment of insulin resistance; TNFα, tumor necrosis factor α; BP, blood pressure; CD, control diet; WTD, Western-type diet; GS, glucose spike.
(TIFF)

S9 Fig The effect of CDDO-Me (3 mg/kg) on gene expression. A, B: Quantitative PCR analysis of the expression of the HMOX1 (A) and GSTP1 (B) mRNAs. * *P* < 0.05, † *P* < 0.01, ‡ *P* < 0.005, and § *P* < 0.001, Bonferroni correction. The data are presented as the means ± SEM. N = 4 rats per group. CD, control diet; WTD, Western-type diet; GS, glucose spike.
(TIFF)

S10 Fig Representative images of DHE staining in the thoracic aortas. Aortas were removed 2 hours after the intraperitoneal administration of glucose (1 g/kg). CDDO-Me (3 mg/kg) reduced the DHE fluorescence intensity in the WTD-GS (+) group. CD, control diet; WTD, Western-type diet; GS, glucose spike.
(TIFF)

S1 Table Assay IDs of the primers and probes used for the quantitative RT-PCR analysis. (DOCX)
