# Supplementary figures and images for: Repeated glucose spikes and insulin resistance synergistically deteriorate endothelial function and bardoxolone methyl ameliorates endothelial dysfunction
Source: PLoS One. 2022 Jan 24;17(1):e0263080. doi: 10.1371/journal.pone.0263080 (PMC8786204; doi:10.1371/journal.pone.0263080)

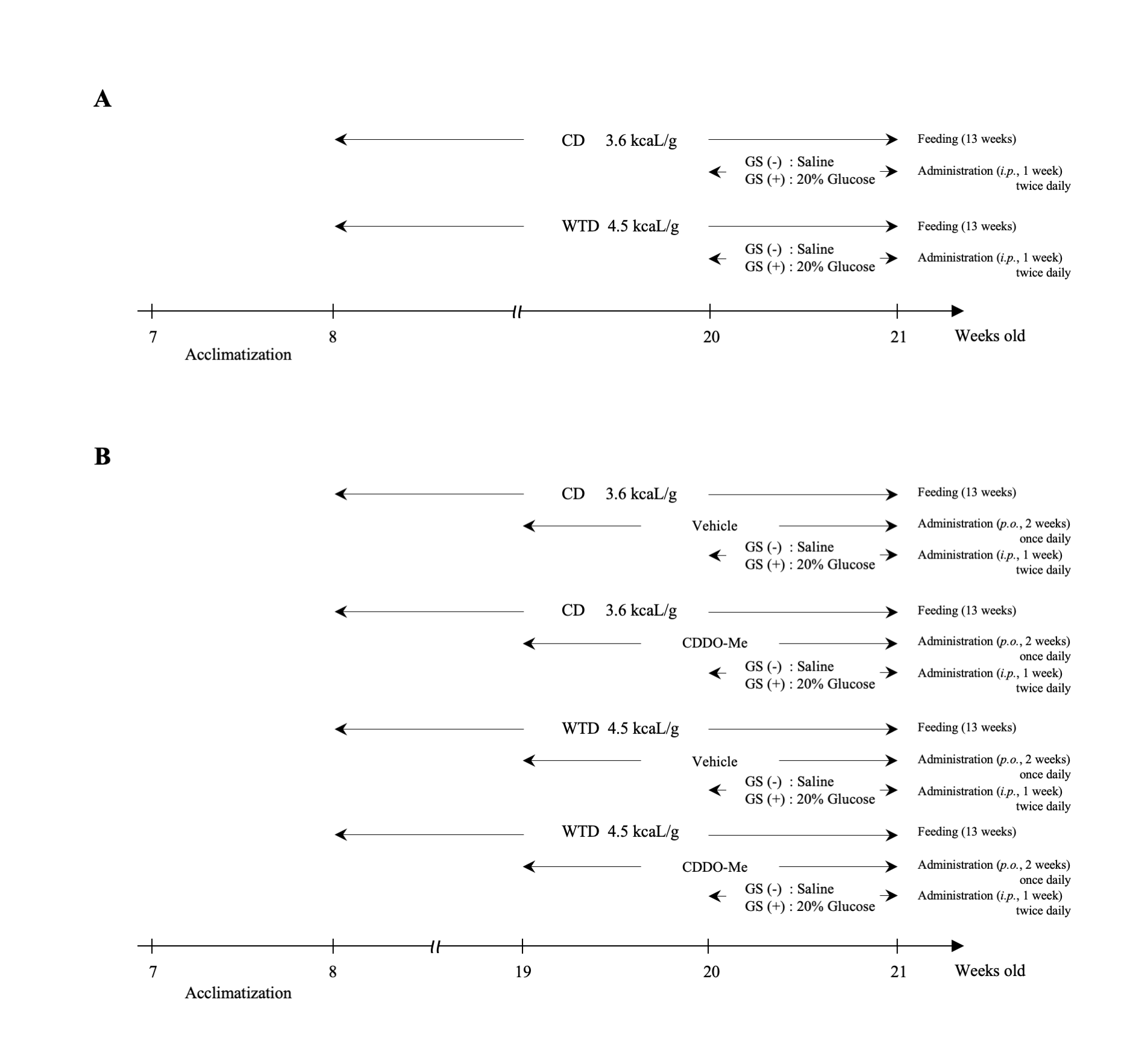

Supplement: S1 Fig — A: First cohort, the comparison of 4 groups: CD-GS (-), CD-GS (+), WTD-GS (-) and WTD-GS (+). Rats were fed a CD or WTD for 13 weeks and administered saline or glucose for 1 week (N = 7 rats per group). Saline and glucose were intraperitoneally administered twice daily for 1 week. B: Second cohort, examination of the effect of CDDO-Me on endothelial function. Rats were fed a CD or WTD for 13 weeks, treated with vehicle or CDDO-Me for 2 weeks and administered saline or glucose for 1 week (N = 4 rats per group). Vehicle (sesame oil) and CDDO-Me were orally administered once daily for 2 weeks. p.o., per os; i.p., intraperitoneal; CD, control diet; WTD, Western-type diet; GS, glucose spike. (TIFF) [file pone.0263080.s001.tiff]

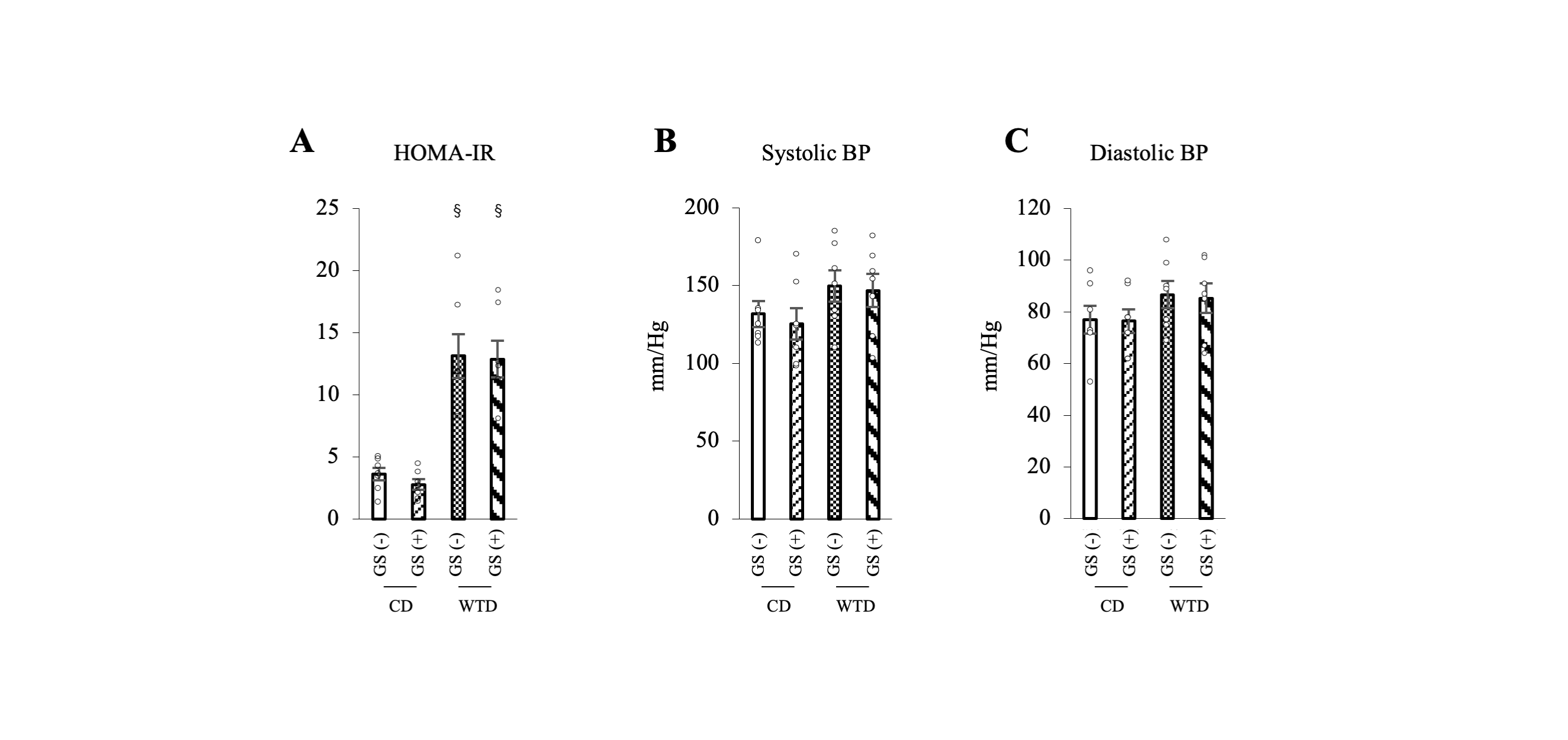

Supplement: S2 Fig — A: HOMA-IR. B-C: Systolic and diastolic blood pressure. § P < 0.001 for the comparison between diet groups, two-way ANOVA. No interaction was detected between diet and GS factors. Data are presented as the means ± SEM. N = 7 rats per group. HOMA-IR, homeostasis model assessment of insulin resistance; BP, blood pressure; CD, control diet; WTD, Western-type diet; GS, glucose spike. (TIFF) [file pone.0263080.s002.tiff]

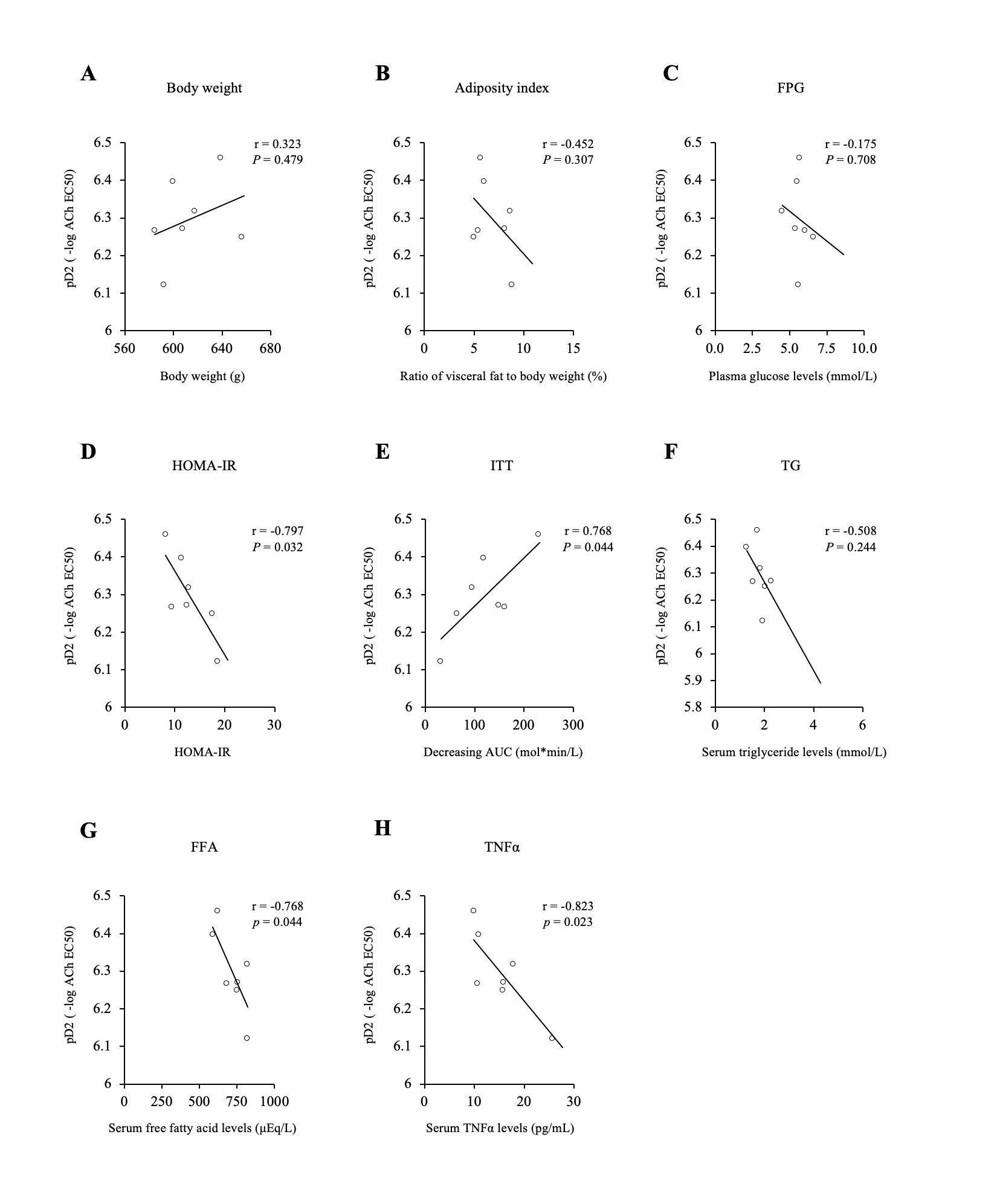

Supplement: S3 Fig — HOMA-IR, serum FFA and TNFα levels were negatively correlated, and the ITT (decreasing AUC) was positively correlated with the pD2 of the WTD-GS (+) group under the 20 mM glucose condition (N = 7). Correlations between body weight (A), adiposity index (B), FPG (C), HOMA-IR (D), ITT (E), serum TG levels (F), serum FFA levels (G) or serum TNFα levels (H) with the pD2 of the WTD-GS (+) group under 20 mM glucose conditions. The adiposity index is the ratio of epididymal, retroperitoneal and mesenteric fat mass to body weight. The ITT is presented as the decreasing glucose AUC. HOMA-IR, homeostasis model assessment of insulin resistance; WTD, Western-type diet; GS, glucose spike; r, Pearson’s correlation coefficient. (TIFF) [file pone.0263080.s003.tiff]

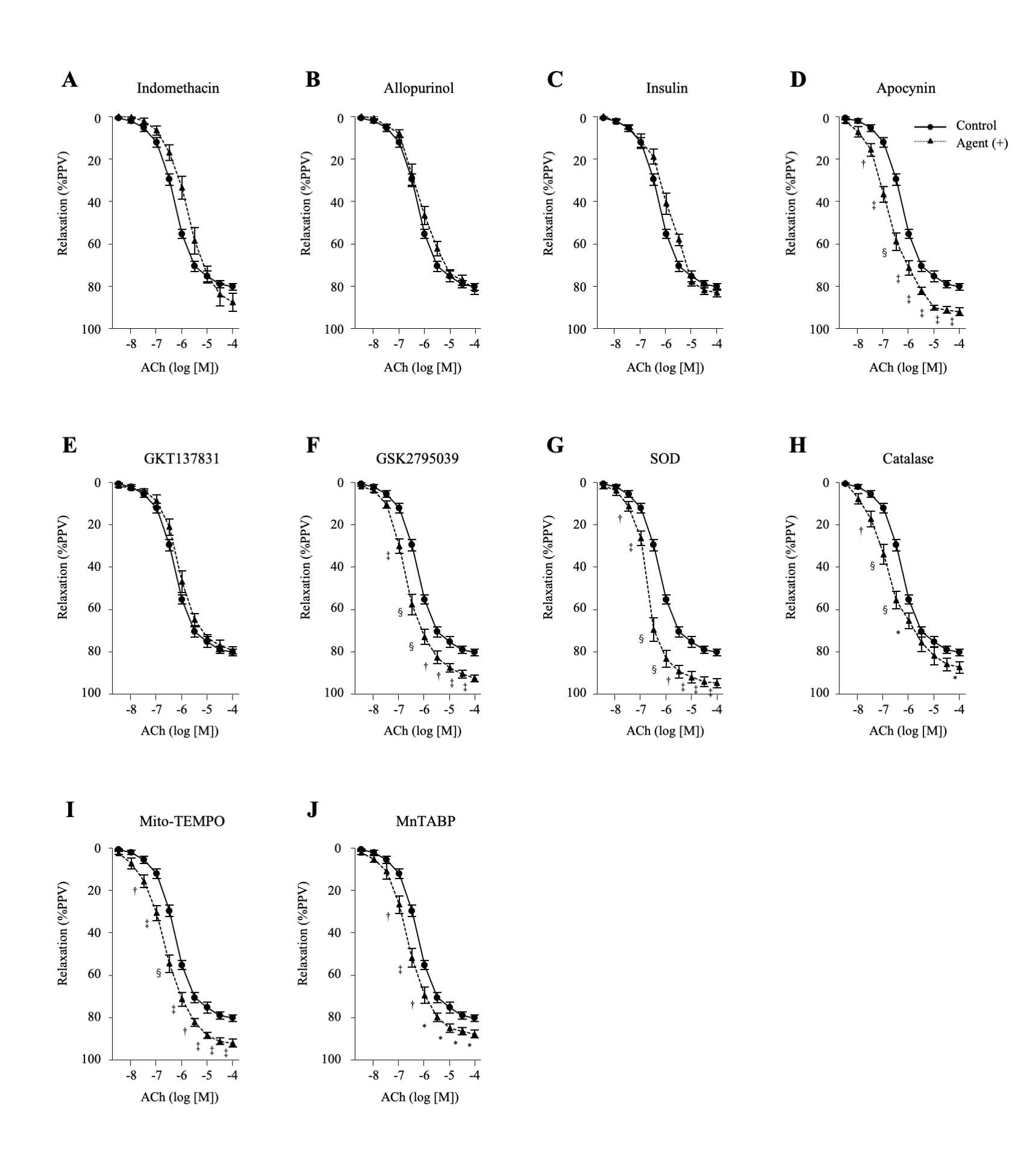

Supplement: S4 Fig — A: Indomethacin, a cyclooxygenase inhibitor; B: allopurinol, a xanthine oxidase inhibitor; C: insulin; D: apocynin, a NADPH oxidase (NOX) inhibitor; E: GKT137831, a NOX1 and 4 inhibitor; F: GSK2795039, a NOX2 inhibitor; G: SOD, superoxide dismutase; H: catalase; I: Mito-TEMPO, a mitochondria-targeted superoxide scavenger; J: MnTABP, a superoxide dismutase mimetic and peroxynitrite selective scavenger. N = 7 (control) or 4 (each agent) rats per group. * P < 0.05, † P < 0.01, ‡ P < 0.005, and § P < 0.001 compared with the control, one-way repeated-measures ANOVA. The data are presented as the means ± SEM; WTD, Western-type diet; GS, glucose spike. (TIFF) [file pone.0263080.s004.tiff]

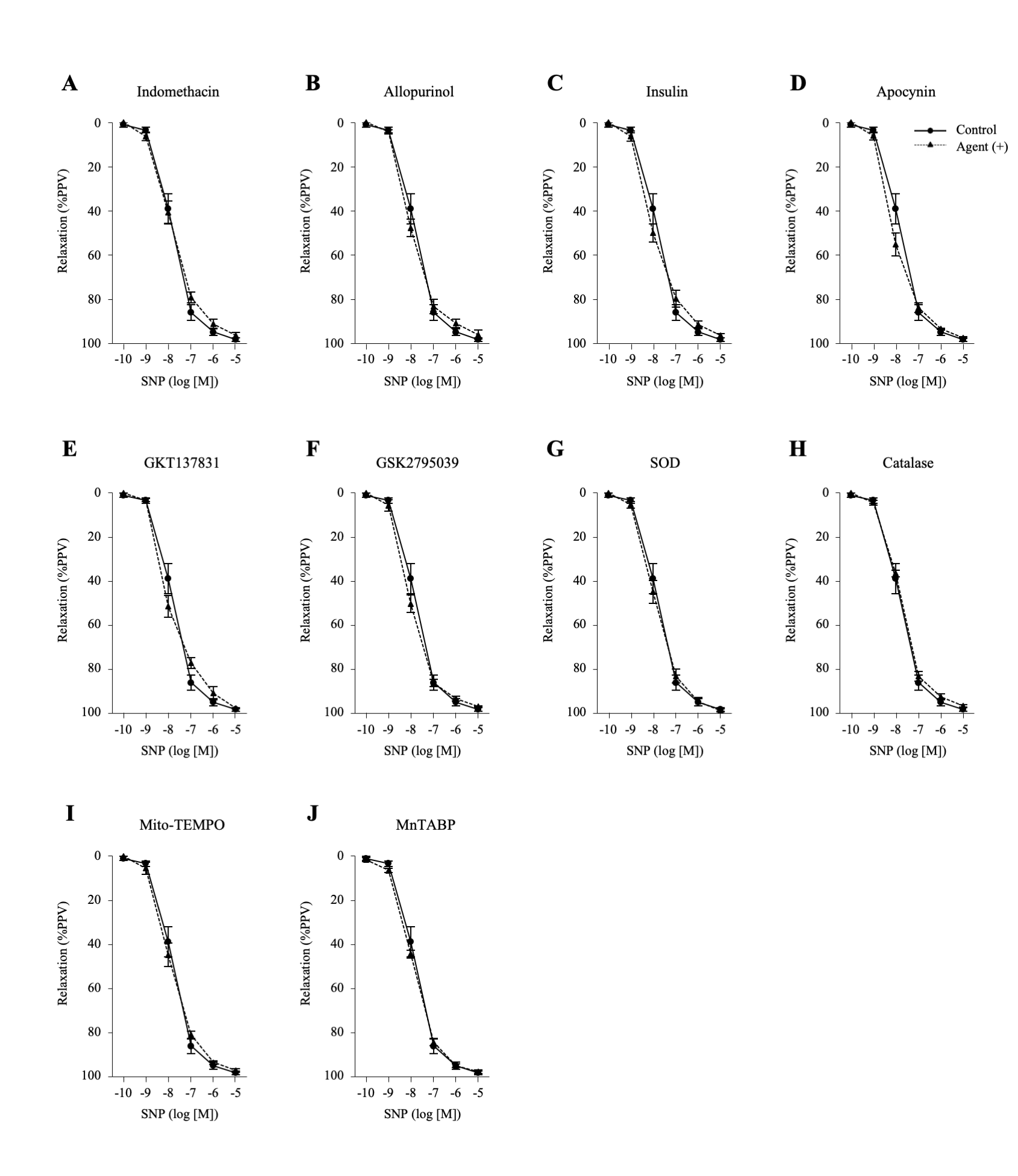

Supplement: S5 Fig — None of the agents altered vascular reactivity to SNP. A: Indomethacin, a cyclooxygenase inhibitor; B: allopurinol, a xanthine oxidase inhibitor; C: insulin; D: apocynin, a NADPH oxidase (NOX) inhibitor; E: GKT137831, a NOX1 and 4 inhibitor; F: GSK2795039, a NOX2 inhibitor; G: SOD, superoxide dismutase; H: catalase; I: Mito-TEMPO, a mitochondria-targeted superoxide scavenger; J: MnTABP, a superoxide dismutase mimetic and peroxynitrite selective scavenger. N = 7 (control) or 4 (each agent) rats per group, one-way repeated-measures ANOVA. The data are presented as the means ± SEM; WTD, Western-type diet; GS, glucose spike. (TIFF) [file pone.0263080.s005.tiff]

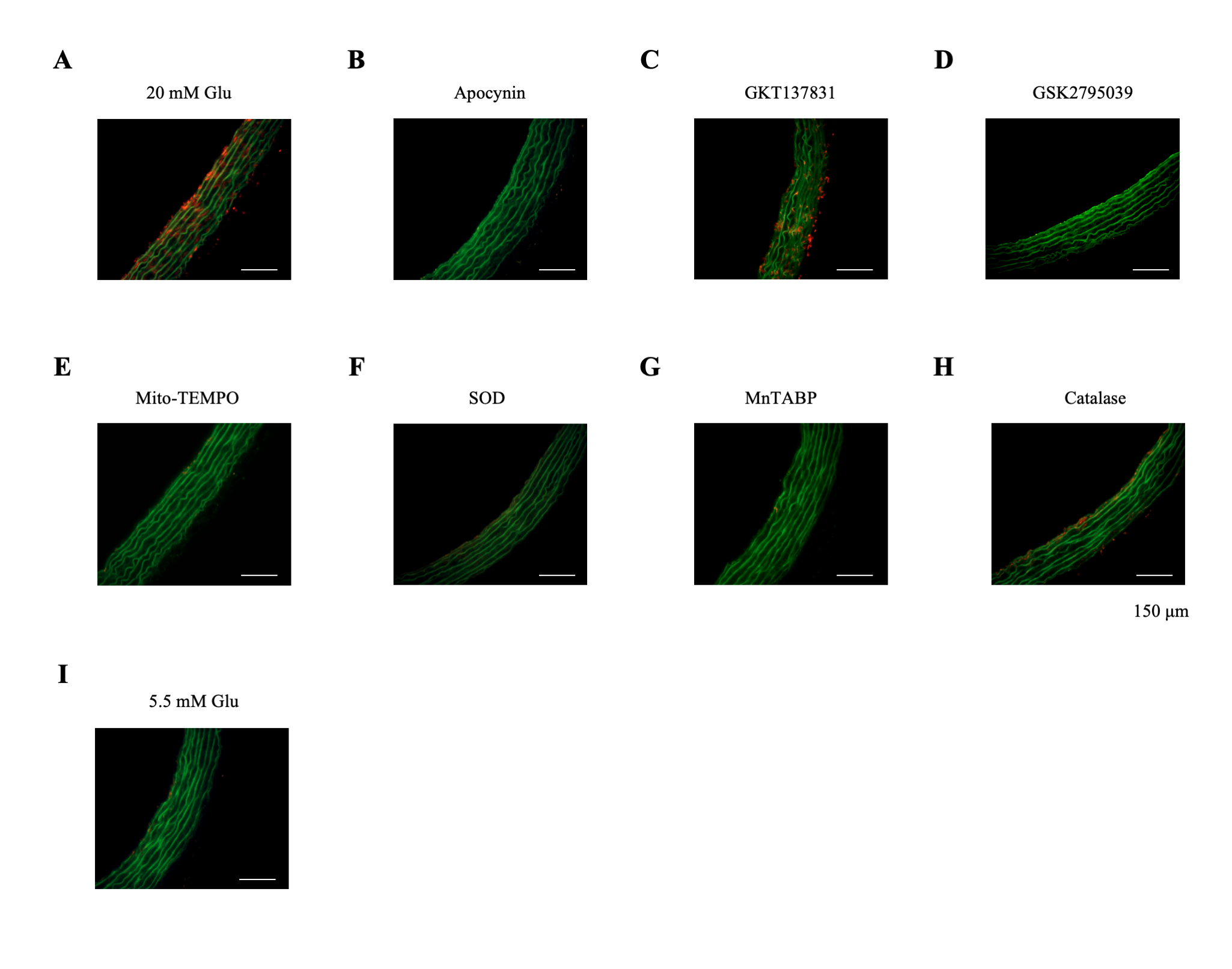

Supplement: S6 Fig — A: 20 mM glucose condition. The aortas were equilibrated in 20 mM glucose for 2 hours without any agents. B-H: The aortas were equilibrated in 20 mM glucose for 2 hours in the presence of apocynin (B), GKT137831 (C), GSK2795039 (D), Mito-TEMPO (E), SOD (F), MnTABP (G) and catalase (H) for 30 minutes. I: 5.5 mM glucose condition. The aortas were equilibrated in 5.5 mM glucose for 2 hours with no agents. Apocynin, an NADPH oxidase (NOX) inhibitor; GKT137831, a NOX1 and 4 inhibitor; GSK2795039, a NOX2 inhibitor; Mito-TEMPO, a mitochondria-targeted superoxide scavenger; SOD, superoxide dismutase; MnTABP, a superoxide dismutase mimetic and peroxynitrite selective scavenger. (TIFF) [file pone.0263080.s006.tiff]

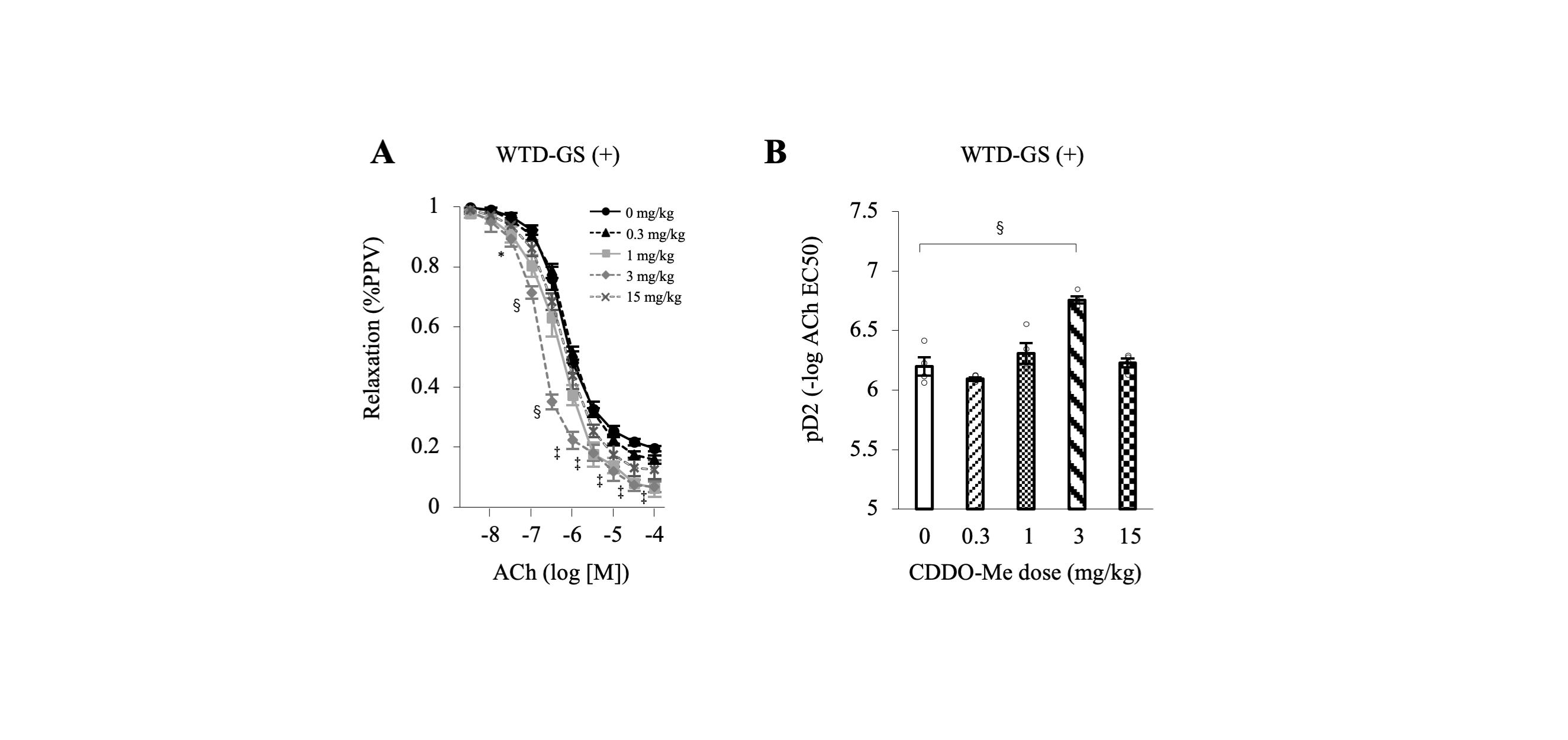

Supplement: S7 Fig — A: Curves of EDR in response to ACh under 20 mM glucose conditions after exposure to 5 doses of CDDO-Me (0 [vehicle], 0.3, 1, 3, and 15 mg/kg). * P < 0.05, ‡ P < 0.005, and § P < 0.001 compared with the 0 mg/kg (vehicle) group, one-way repeated-measures ANOVA. B: Vascular sensitivity, plotted as pD2 (- log of the half-maximal effective concentration [EC50]) of ACh. § P < 0.001 compared with the 0 mg/kg (vehicle) group, Dunnett’s test. The data are presented as the means ± SEM. N = 4 rats per group. WTD, Western-type diet; GS, glucose spike. (TIFF) [file pone.0263080.s007.tiff]

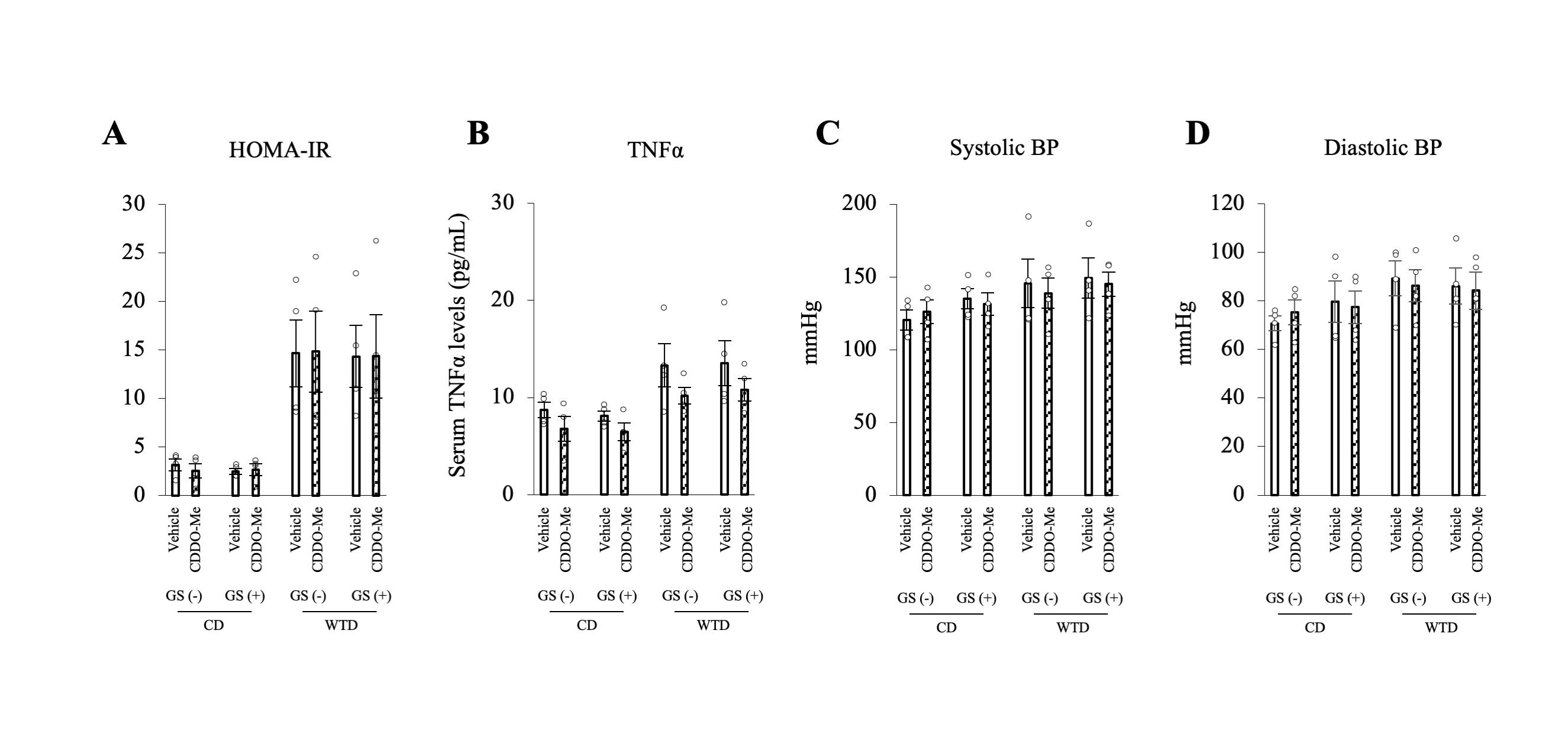

Supplement: S8 Fig — A-D: HOMA-IR (A), serum TNFα levels (B), systolic blood pressure (C) and diastolic blood pressure (D). No significant differences were observed between the vehicle and CDDO-Me in each group using the Bonferroni correction. The data are presented as the means ± SEM. N = 4 rats per group. HOMA-IR, homeostasis model assessment of insulin resistance; TNFα, tumor necrosis factor α; BP, blood pressure; CD, control diet; WTD, Western-type diet; GS, glucose spike. (TIFF) [file pone.0263080.s008.tiff]

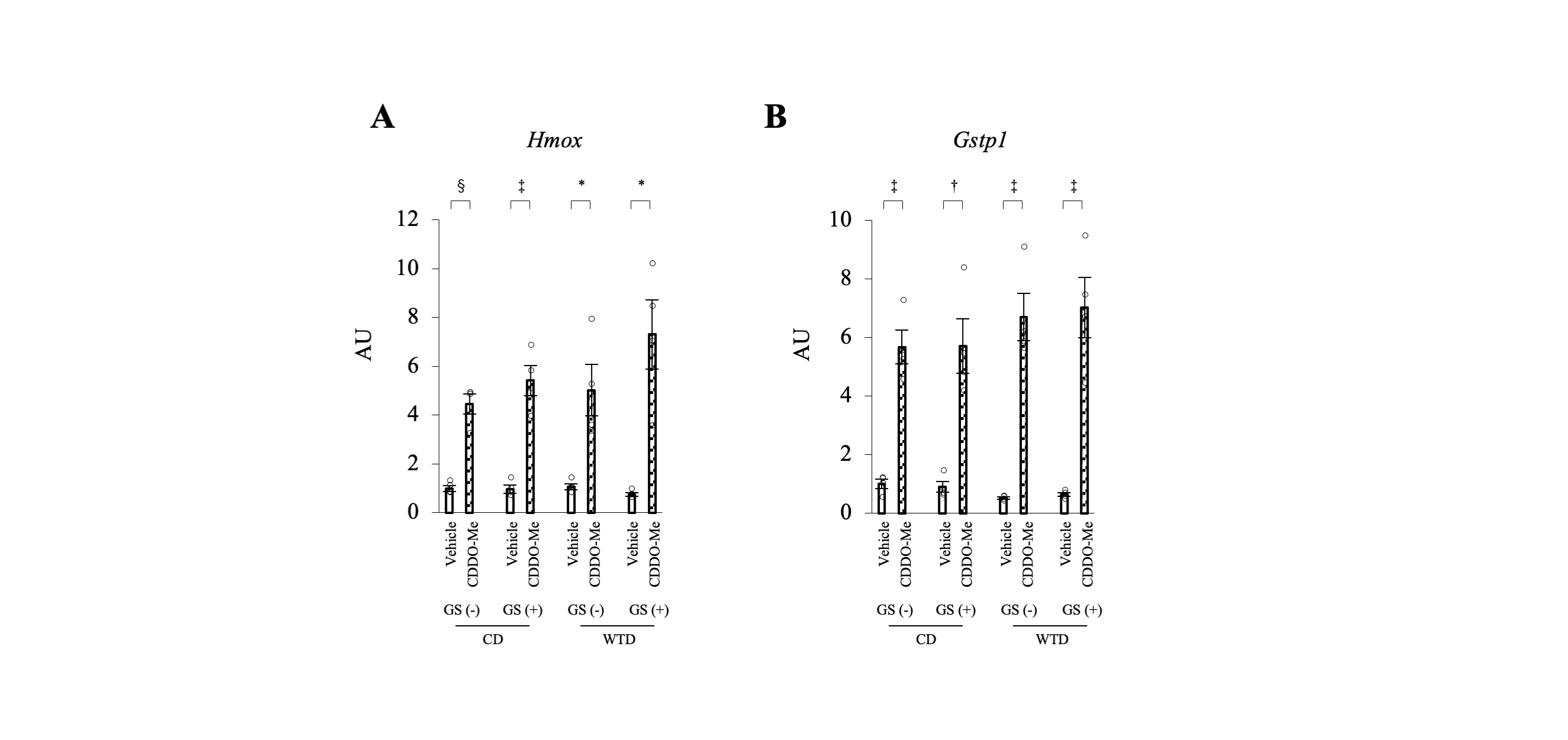

Supplement: S9 Fig — A, B: Quantitative PCR analysis of the expression of the HMOX1 (A) and GSTP1 (B) mRNAs. * P < 0.05, † P < 0.01, ‡ P < 0.005, and § P < 0.001, Bonferroni correction. The data are presented as the means ± SEM. N = 4 rats per group. CD, control diet; WTD, Western-type diet; GS, glucose spike. (TIFF) [file pone.0263080.s009.tiff]

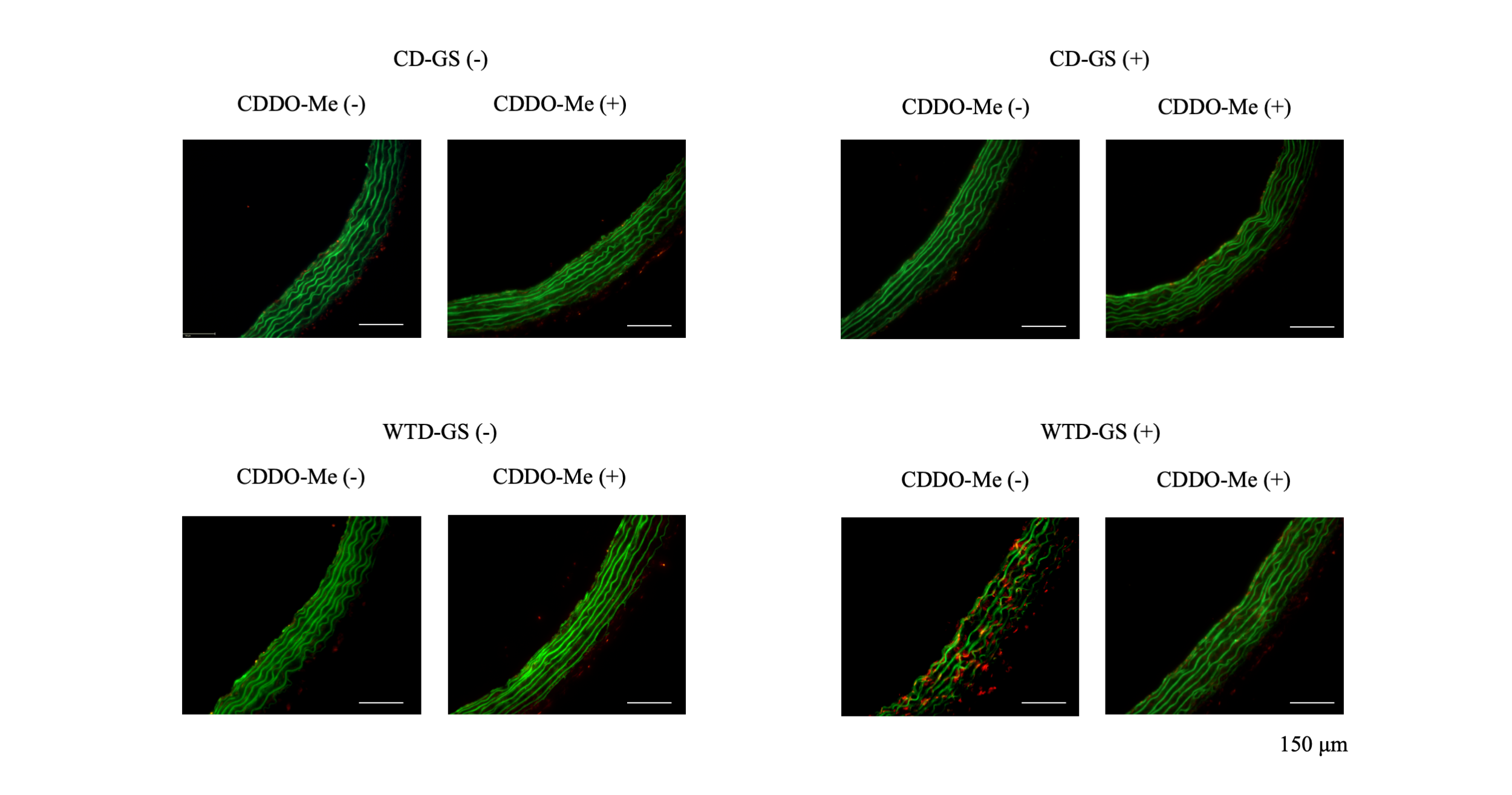

Supplement: S10 Fig — Aortas were removed 2 hours after the intraperitoneal administration of glucose (1 g/kg). CDDO-Me (3 mg/kg) reduced the DHE fluorescence intensity in the WTD-GS (+) group. CD, control diet; WTD, Western-type diet; GS, glucose spike. (TIFF) [file pone.0263080.s010.tiff]
