# Supplementary material for: Repeated glucose spikes and insulin resistance synergistically deteriorate endothelial function and bardoxolone methyl ameliorates endothelial dysfunction
Source: PLoS One. 2022 Jan 24;17(1):e0263080. doi: 10.1371/journal.pone.0263080 (PMC8786204; doi:10.1371/journal.pone.0263080)
Supplement: S1 Table — (DOCX) [file pone.0263080.s011.docx]

| **S1 Table.** Assay IDs of the primers and probes used for the quantitative RT-PCR analysis. | |
| --- | --- |
| Gene | Assay ID |
| *Nox1* | Rn00586652_m1 |
| *Nox2* (*Cybb*) | Rn00576710_m1 |
| *p47phox* (*Ncf1*) | Rn00586945_m1 |
| *Nox4* | Rn00585380_m1 |
| *Sod1* | Rn00566938_m1 |
| *Sod2* | Rn00690588_g1 |
| *Cat* | Rn00560930_m1 |
| *Gpx1* | Rn00577994_g1 |
| *Tnf* | Rn99999017_m1 |
| *Il1b* | Rn00580432_m1 |
| *Vcam1* | Rn00563627_m1 |
